# Nimodipine protects Schwann and neuronal cells from cell death induced by cisplatin without affecting cancer cells

**DOI:** 10.1038/s41598-025-06854-5

**Published:** 2025-06-25

**Authors:** Sandra Leisz, Saskia Fritzsche, Christian Strauss, Maximilian Scheer, Christian Scheller

**Affiliations:** 1 Department of Neurosurgery, Medical Faculty, Martin Luther University Halle-Wittenberg, Ernst-Grube-Str. 40, 06120 Halle (Saale), Germany; 2 Department of Neurosugery, University Hospital Heidelberg, Im Neuenheimer Feld 400, 69120 Heidelberg, Germany

**Keywords:** Nimodipine, Neuropathy, Cisplatin, Neuroprotection, Schwann cells, Neuronal cells, Peripheral nervous system, Cancer therapy, Peripheral neuropathies, Oral cancer, Non-small-cell lung cancer, Ovarian cancer

## Abstract

Cisplatin is a well-established drug for the treatment of solid tumors. One of the most common side effects is neurotoxicity and peripheral neuropathy, which affects patients’ quality of life. In previous studies, a protective effect of nimodipine on neuronal cell stress was demonstrated. Therefore, the objective of this study was to examine the impact of nimodipine on cisplatin-treated Schwann cells, neuronal cells, and tumor cells. Schwann and neuronal cells were used to investigate the neuroprotective effect of nimodipine, as well as the cancer cell lines A549, SAS and SKOV-3 to determine the effect on tumor cells. Cell death was measured using extracellular lactate dehydrogenase activity and propidium iodide staining. In addition, the protein level of the LIM-domain only four protein and the activation of known interacting anti-apoptotic pathways were analyzed. The cytotoxic effect of cisplatin was reduced by up to 23.6% in neuronal cells (*p* ≤ 0.0001) and up to 30.6% in Schwann cells (*p* ≤ 0.05) by nimodipine pre-treatment. However, no decrease in apoptosis could be shown in the cancer cells. Nimodipine-dependent activation of anti-apoptotic signaling pathways was detectable in Schwann cells and neuronal cells, whereas the opposite effect could be demonstrated in the cancer cells. In conclusion, the treatment with nimodipine may represent a new approach against neurotoxically side effects in cisplatin chemotherapy.

**Supplementary Information:**

The online version contains supplementary material available at 10.1038/s41598-025-06854-5.

## Introduction

Nimodipine (NIMO) is a 1, 4-dihydropyridine-type calcium channel antagonist that exerts its effect by binding to the alpha 1 subunit of the L-type voltage-gated calcium channel by negative allosteric inhibition^1–3^. In smooth muscle, this prevents the influx of calcium from extracellular to intracellular and thus relaxes the vessels^3^. L-type calcium channels, the Ca_v_1 subfamily, are expressed in various tissues^2,4^. The alpha 1c (Ca_v_1.2) and 1d subunit (Ca_v_1.3) were primarily detected in neuronal tissue^5^. Developed as a drug for treatment against hypertension, nimodipine is nowadays used for the prophylaxis and therapy of aneurysmal subarachnoid hemorrhages (aSAH)^1,6^ due to its lipophilic properties and thus its good cerebrospinal fluid penetrability compared to other substances of its class^2^. In this process improved cerebral blood flow and oxygenation can be achieved by counteracting the formation of vasospasms caused by blood degradation products^6^.

However, over the last decades, the calcium channel antagonists have been shown to have a neuroprotective effect on neuronal, Schwann and auditory cells^7,8^, as well as in surgery-like in vitro models^9,10^ and led to a reduced amount of hearing loss after vestibular schwannoma surgery^11,12^. The activation of anti-apoptotic signaling pathways through phosphorylation of Ak strain transforming/protein kinase B (AKT)^8^ and activation of the janus kinase/signal transducer and activator of transcription (JAK/STAT) signaling pathway^13,14^ underlines the neuroprotective effect on neuronal and Schwann cells, as well as on auditory hair cells. In addition, NIMO is suggested to reduce calcium homeostasis imbalances and protect cells from calcium overload, as calcium plays a fundamental role in neuronal cell function and general cell survival.

Disruption of calcium homeostasis also occurs during treatment with platinum-based chemotherapy, leading to calcium overload and apoptosis^15^. Despite its approval in 1978^16^, cisplatin (CIS) is still the standard therapy for many solid tumors, including testicular, ovarian, bladder, non-small cell lung carcinoma and squamous cell carcinomas of the head and neck^16–18^. Due to its molecular structure, it is able to penetrate the plasma membrane^16,19^ and leads to the formation of cross-links within the DNA, preventing transcription and resulting in cell death^20^. In addition, it leads to disruption of the calcium balance and mitochondrial dysfunction leading to increased formation of reactive oxygen species (ROS) and initiation of apoptosis^20^. Since the effect of CIS is not only limited to cancer cells, time and dose-limiting side effects occur more frequently during chemotherapy, which not only reduce the obtained benefit of the therapy, but also significantly impair the quality of life of patients undergoing chemotherapy. Most frequently, the therapy leads to neurotoxicity^20,21^, ototoxicity and nephrotoxicity^22,23^, resulting in chemotherapy-induced peripheral neuropathy (CIPN), hearing loss and nephropathy. Initial symptoms in patients with CIPN present as sensory deficits in the distal limbs, caused by neuroinflammation, DNA damage and axon degeneration^20,21,24^.

Oxidative stress caused by ROS generation by CIS also leads to nitration of the LIM-domain only four protein (LMO4)^25^. LMO4 is known to be a transcriptional regulator^26^ that plays an important role in the regulation of neuronal development, activity and survival, depending on calcium concentration^27^ and by forming transcriptional complexes with e.g. cAMP response element-binding protein (CREB)^28^.In addition, it influences the calcium balance by regulating the expression of ryanodine 2 receptors^28,29^ and leads to the activation of anti-apoptotic signaling pathways by interacting with AKT^30^.

Despite increased attempts to develop a therapy to prevent the side effects caused by CIS, there is still no established standard therapy^21,22,31^. Therefore, the aim of this study refers to analyze the protective effect of NIMO, with a more detailed investigation into the molecular mechanisms involved. Furthermore, the question is determining whether the protective effects of the calcium channel antagonist are limited to neuronal cells or whether the tumor is also protected.

## Materials and methods

### Cell lines

The neuronal cell line RN33B (#CRL-2825, neurons, rat) and the cell line SW10 (#CRL-2766, Schwann cells, mouse) were obtained from the American Type Culture Collection (Manassas, VA, USA). The human cancer cell lines A549 (NSCLC, non-small cell lung cancer), SAS (squamous cell carcinoma of the tongue) and SKOV-3 (ovarian cancer) were kindly provided by Barbara Seliger from the Institute of Medical Immunology (Martin Luther University Halle-Wittenberg, Halle (Saale), Germany). The SW10, A549 and SAS cells were cultivated in Dulbecco’s Modified Eagle Medium (DMEM) (Thermo Fisher Scientific, Waltham, MA, USA). The RN33B cells were cultured in DMEM/F12 (1:1, Thermo Fisher Scientific, Waltham, MA, USA) and the SKOV-3 cells were cultured in RPMI. Each cell medium was supplemented with 1% penicillin–streptomycin (10,000 U/mL penicillin/10,000 µg/mL streptomycin) (Gibco, Thermo Fisher Scientific, Waltham, MA, USA) and 10% fetal bovine serum (FBS, Gibco, Thermo Fisher Scientific, Waltham, MA, USA). The cells were cultured in 75 cm^2^ cell culture flasks (Sarstedt, Nümbrecht, Germany) in a humidified atmosphere containing 5% CO_2_ at 37 °C.

### NIMO and CIS treatment

A total of 5 × 10^4^ cells of each cell line were seeded in 24-well plates (Techno Plastic Products, TPP, Trasadingen, Switzerland) and treated 24 h prior to CIS application with 10 µM and 20 µM NIMO diluted in absolute ethanol (EtOH). The equal amount of EtOH was added to untreated controls, resulting in a final concentration of 0.1% (solvent control). The NIMO solutions and treated cells were protected from light exposure. CIS (Sigma-Aldrich, Merck, Darmstadt, Germany) was solved in sterile 0.9% NaCl (Braun, Melsungen, Germany) and then added to each cell culture in a final concentration of 10 µM and 20 µM, while the same amount of NIMO as before was added again. An overview of the treatment scheme and experimental setup is provided in Figure S1.

### Cytotoxicity measurement

Following a 24- and 48-h incubation period, cytotoxicity was quantified by the activity of lactate dehydrogenase (LDH) as a marker of cell death utilizing the Cytotoxicity Detection Kit (Roche, Basel, Switzerland) according to the manufacturer’s instructions. In brief, 100 µL of cell culture supernatant in triplicate per sample and 100 µL of reaction mixture were incubated for 30 min in the dark. The absorbance was then measured at 492 nm using a Tecan Reader F2000 Pro (Tecan, Männedorf, Switzerland) at four defined points within the wells. The absorbance of cells lysed with 2% Triton X-100 (Carl Roth, Karlsruhe, Germany) served as a positive control representing 100% cell death. The medium signal without cells served as a background signal. The calculation of the cell death rate was performed as described before^8,10^ and calculated with the following formula:

$$cell\;death = \frac{{(OD_{{cells}} - OD_{{medium}} )}}{{(OD_{{lysis}} - OD_{{medium}} )}} \times 100\% \;\;\;{\text{OD}} - {\text{optical density}}$$

### Live cell imaging

A total of 5 × 10^4^ cells were seeded in 24-well plates (Techno Plastic Products, TPP, Trasadingen, Switzerland) and treated with 10 µM or 20 µM CIS with or without pre-treatment with 10 µM or 20 µM NIMO as described in Section "NIMO and CIS treatment". Microscopic images were taken 24 h after treatment. Cells were stained with propidium iodide (PI) (Thermo Fisher Scientific, Waltham, MA, USA) and NucBlue™ Live Cell Stain ReadyProbes reagent (Thermo Fisher Scientific, Waltham, MA, USA) in accordance with the manufacturer’s instructions. Subsequently, the cells were washed with Dulbecco’s phosphate buffered saline (PBS, Gibco, Thermo Fisher Scientific, Waltham, MA, USA), 1 mL FluoroBrite™ DMEM (Gibco, Thermo Fisher Scientific, Waltham, MA, USA) was added and the cells were imaged using a Keyence BZ-800E microscope (Keyence, Neu-Isenburg, Germany). The quantification of cells stained with NucBlue™ and PI was conducted using the IdentifyPrimaryObjects function of the CellProfiler software (version 4.2.4, Broad Institute, Cambridge, MA, USA).

### Protein analysis

The cells were seeded in 100 mm × 21 mm petri dishes (Techno Plastic Products, TPP, Trasadingen, Switzerland) and treated with 10 µM and 20 µM CIS with or without pre-treatment with 10 µM or 20 µM NIMO. Following a 24-h incubation, the cells were washed twice with ice-cold PBS and harvested with PBS containing protease and phosphatase inhibitors (Pierce, Thermo Fisher Scientific, Waltham, MA, USA). Proteins were extracted with 1 × Lithium dodecyl sulphate (LDS) sample buffer (Invitrogen, Thermo Fisher Scientific, Waltham, MA, USA) followed by heating at 70 °C for 10 min. The protein concentration was measured using the Pierce BCA Protein Assay Kit (Thermo Fisher Scientific, Waltham, MA USA) following the manufacturer’s instructions. Furthermore, 5% β-mercaptoethanol (Carl Roth, Karlsruhe, Germany) and 1 × LDS sample buffer were added to the proteins, which were then heated at 70 °C for 10 min again.

The separation of proteins was conducted via sodium dodecyl sulphate polyacrylamide gel electrophoresis (SDS PAGE) using NuPAGE™ 4–12%, Bis–Tris, 1.5 mm, Mini-Protein-Gels and NuPAGE™ MES SDS Running Buffer (both obtained from Thermo Fisher Scientific, Waltham, MA, USA). The pre-stained protein ladder (PageRuler, #26,616, Thermo Fisher Scientific, Waltham, MA, USA) was utilized as a molecular weight marker. Subsequently, the proteins were blotted onto 0.2 µm or 0.45 µm nitrocellulose membranes (Amersham, GE, Healthcare, Freiburg, Germany), depending on the molecular weight of the proteins, followed by Ponceau S staining (0.1% Ponceau S, 3% trichloroacetic acid and 3% sulfosalicylic acid).

The membranes were blocked with 5% nonfat dry milk (Carl Roth, Karlsruhe, Germany) in Tris-buffered saline (TBS) containing 0.1% Tween (TBS-T, Sigma-Aldrich, St. Louis, Missouri, USA). The primary antibodies (Table S1) were added and incubated overnight at 4 °C. Following five time washing with TBS-T, the secondary antibodies were added for 60 min at room temperature.

The membranes were developed using Pierce ECL Western Blotting Substrate (Thermo Fisher Scientific, Waltham, MA USA) and the signals were detected using a charge coupled device (CCD) camera (ImageQuant LAS4000, GE Healthcare, Freiburg, Germany). Band intensity was quantified using ImageQuant TL software version 3.0 (GE Healthcare, Freiburg, Germany). The protein level of glyceraldehyde-3-phosphate dehydrogenase (GAPDH) was used as a loading control for each blot.

### Statistical analysis

Data analyses were performed using Excel (version Microsoft Office Professional Plus 2016, Microsoft Corporation, Redmond, WA, USA) and GraphPad Prism 10.4.1 (GraphPad Software Inc., San Diego, CA, USA).

A one-way ANOVA followed by a Tukey’s multiple comparison test was conducted for all cell lines. The figures represent the mean and standard deviation (SD) as indicated in the corresponding figure legends. At least three independent biological replicates were performed for each experiment.

## Results

### NIMO pre-treatment leads to decreased CIS-induced cell death rate in Schwann cells and neuronal cells

**Fig. 1:**
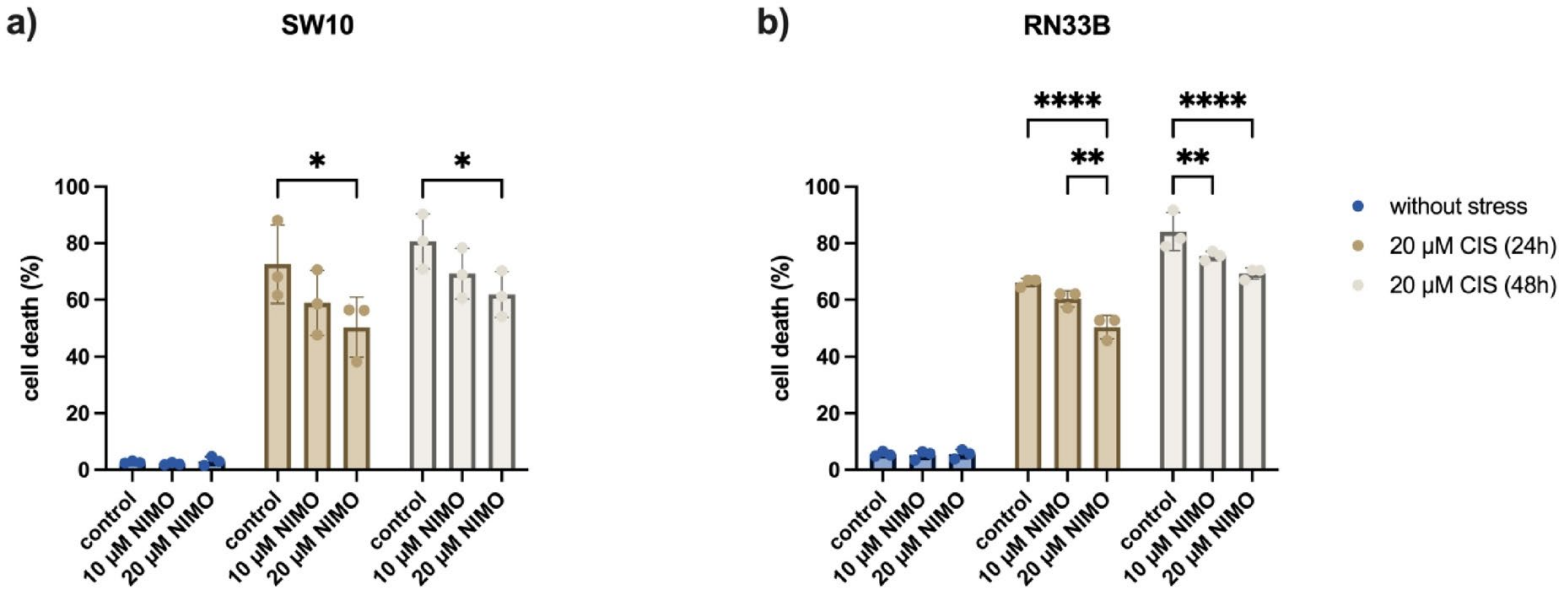
Reduction of CIS-induced cell death by nimodipine pre-treatment in Schwann cells and neuronal cells. LDH activity in the supernatant of SW10 (**a**) and RN33B (**b**) cells was measured 24 h and 48 h after stress induction with 20 μM CIS, without (= solvent control, EtOH) and with prior pre-treatment of NIMO (10 μM and 20 μM), respectively**.** The mean values and SDs of three independent biological replicates are shown in the graphs. *p ≤ 0.05; **p ≤ 0.01; ****p ≤ 0.0001.

**Fig. 2:**
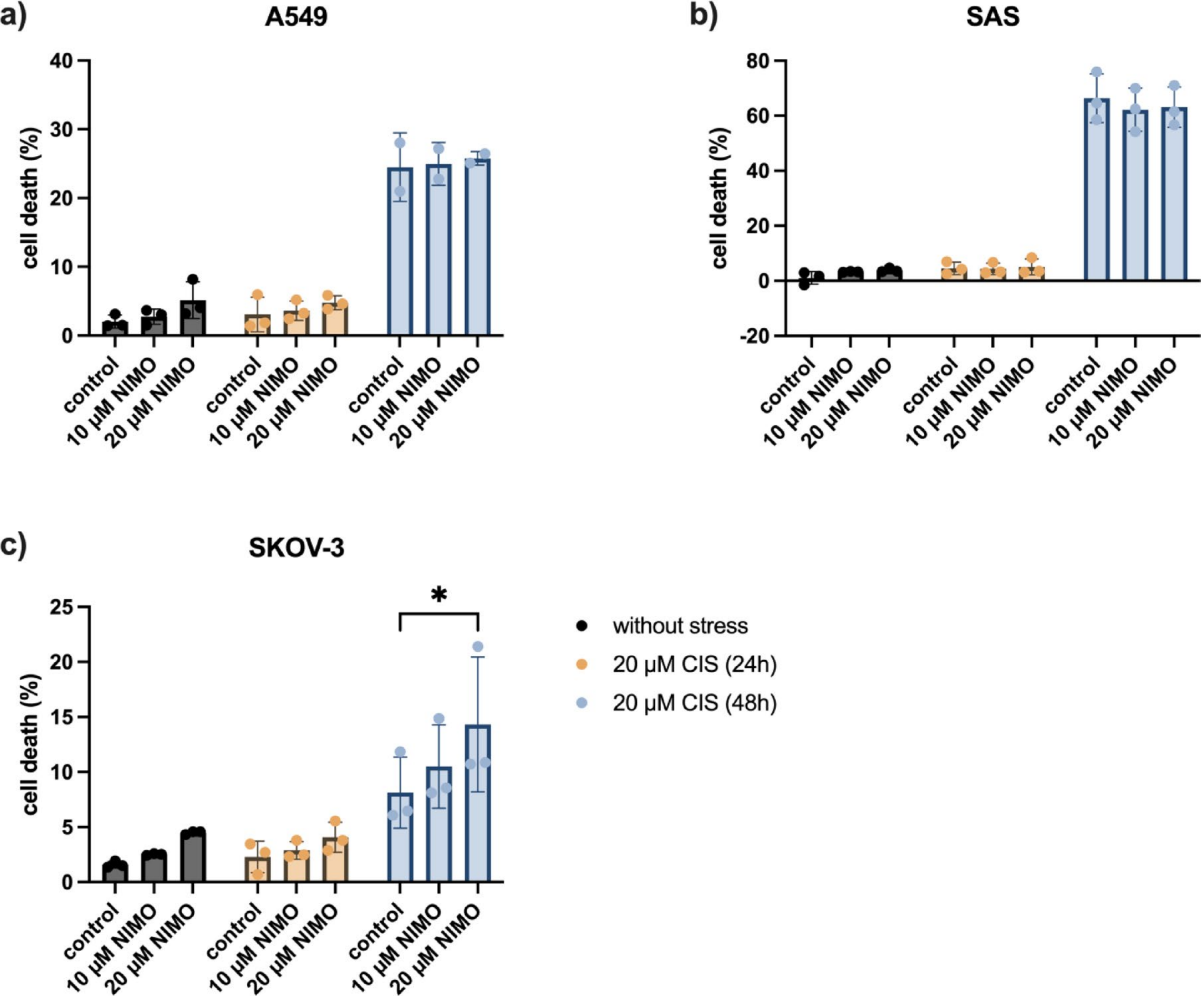
Influence of NIMO pre-treatment on cancer cell lines after 24h and 48h of CIS treatment. LDH activity in the supernatant of A549 (**a**), SAS (**b**) and SKOV-3 cells (**c**) was measured 24 and 48 h after stress induction with 20 μM CIS, without (solvent control, EtOH) or with prior pre-treatment with NIMO (10 and 20 μM). The mean values and SDs of three independent biological replicates are shown in the figures. *p ≤ 0.05.

To measure the cytotoxicity caused by treatment with 20 µM CIS and the effect of pre-treatment with 10 µM and 20 µM NIMO, extracellular lactate dehydrogenase levels were used as a marker of cell death. No relevant change in the cell death rate was measured after treatment with NIMO in both cell lines without stress (Fig. 1). After adding 20 µM CIS a significant increase in dead cells in SW10 up to 72.6% ± 9.8% (Fig. 1a) and in RN33B cell line up to 66.1% ± 1.1% (Fig. 1b) after 24 h was detected. For both, SW10 and RN33B cell line, NIMO induced a decrease in cell death rate under CIS stress. In comparison to the control group (EtOH) pre-treatment with NIMO showed a reduction to 59.0% ± 8.1% (10 µM NIMO, non-significant (n.s.)) and 50.4% ± 7.5% (20 µM NIMO, *p* ≤ 0.05) in SW10 cells (Fig. 2a). NIMO pre-treatment in RN33B cells lead to a significant decrease in cell death rate to 60.4% ± 2.0% (10 µM NIMO, *p* ≤ 0.05) and to 50.5% ± 2.9% (20 µM NIMO, *p* ≤ 0.0001) when comparing to solvent control (EtOH, Fig. 1b). After 48 h, the cell death rate increased to 80.7% ± 7.9% in the SW10 cell line and to 84.1% ± 5.5% in the RN33B cell line after treatment with 20 µM CIS. NIMO pretreatment led to a reduction of the cell death rate to 69.3% ± 7.3% (10 µM NIMO, n.s.) and with the higher NIMO concentration to 61.9% ± 6.6% (20 µM NIMO, *p* ≤ 0.05) in Schwann cells. Furthermore, a reduced cell death was determined by NIMO pretreatment to 75.5% ± 1.3% (10 µM NIMO, *p* ≤ 0.01) and 69.3% ± 1.6% (20 µM NIMO, *p* ≤ 0.0001) in the RN33B cells. Further multiple statistical analysis between all groups are shown in Table S2 and S3.

### NIMO pre-treatment did not protect the cancer cells to CIS application

The cancer cell lines A549 (non-small cell lung cancer), SAS (squamous cell carcinoma of the tongue) and SKOV-3 (ovarian cancer) cells did not show a significant increase in cell death after 24 h of treatment with 20 µM CIS (Fig. 2). Therefore, an additional measurement was performed after 48 h to investigate the effect of NIMO under CIS-induced stress. After 48 h, treatment with 20 µM CIS led to an increase in the cell death rate up to 24.5% ± 2.9% (solvent control) in A549 cells and up to 66.4% ± 6.3% (solvent control) in SAS cell line (Fig. 2a,b). After 48 h, we observed no significantly altered cell death level under 10 µM (25.0% ± 1.8%; n.s.) and 20 µM NIMO (25.8% ± 0.6, n.s.) in A549 cells (Fig. 2a) in comparison to CIS-treated control cells. For SAS cells there was also no significant effect detectable with pre-treatment of 10 µM NIMO (62.2% ± 5.5%, n.s.) and 20 µM NIMO (63.1% ± 5.2, n.s.) under CIS administration (Fig. 2b). The analysis of SKOV-3 cells after NIMO pre-treatment and stress induction with CIS demonstrated a tendency of increase in cell death induced by NIMO from 8.1% ± 2.3% (solvent control) to 10.5% ± 2.7% (10 µM NIMO, n.s.). A significant increase to 14.3% ± 4.3% (*p* ≤ 0.05) induced by treatment with 20 µM NIMO compared to the solvent control was detected at 48 h (Fig. 2c). Additionally, without stress and with a low cell death rate under 10%, there was a tendency to increase the cytotoxic effect on the SKOV-3 cells from 1.6% ± 0.2% (solvent control) to 2.5% ± 0.1% (10 µM NIMO, n.s.) and to 4.5% ± 0.1% (20 µM NIMO, n.s.). A slight increase in cell death under CIS from 2.3% ± 1.2% to 2.9% ± 0.7% (10 µM NIMO, n.s.) and to 4.1% ± 1.1% (20 µM NIMO, n.s.) was detectable after 24 h (Fig. 2c). Further multiple statistical comparison between all groups can be found in Table S4, S5 and S6.

### NIMO treatment affects cell morphology and cell viability during CIS application

**Fig. 3:**
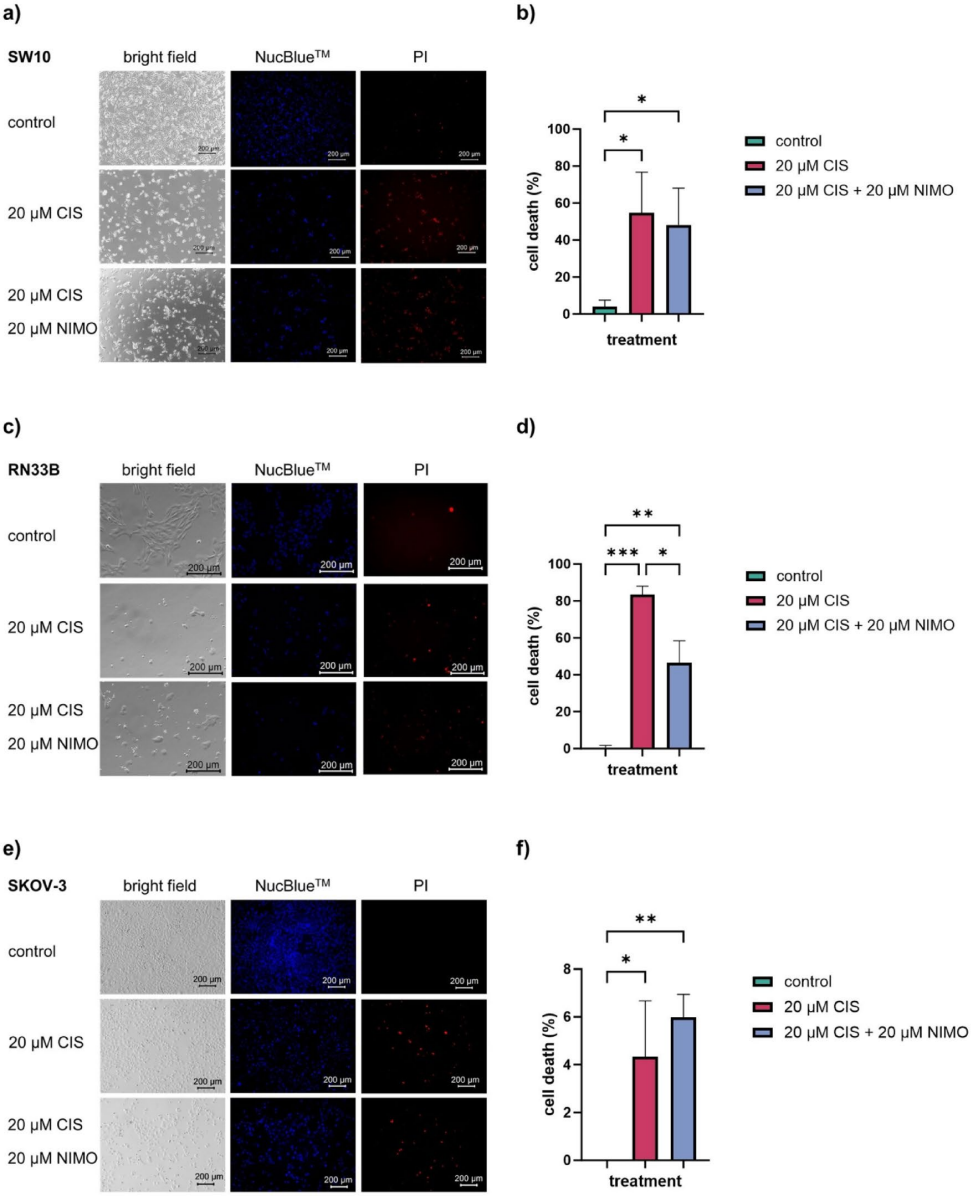
Microscopic images including cell death quantification of Schwann cells, neuronal cells and ovarian cancer cell line after 48h stress induction. Microscopic images and its quantification of the cell lines SW10 (**a**, **b**), RN33B (**c**, **d**) and SKOV-3 (**e**, **f**); top row control, middle row with CIS treatment, bottom row under pre-treatment with NIMO. The bright field image is shown in the left column, NucBlue™ in the middle column and PI staining in right column (**a**, **c**, **e**). The images are representative of three independent biological replicates, while the graphs show the mean and SDs of all replicates after quantification (**b**, **d**, b). *p ≤ 0.05; **p ≤ 0.01; ***p ≤ 0.001.

To analyze the cell morphological alterations of CIS treatment and the combined treatment with NIMO in more detail, the SW10, RN33B and SKOV-3 cells were microscopically examined. Furthermore, the cells were stained with NucBlue™ and PI to visualize and quantify the death cells. The microscopic images of the cell line SW10 (Fig. 3a) showed morphological changes under CIS treatment. In addition, the number of cells and the cell aggregation decreases considerably (bright field). With NIMO pre-treatment, a further increase in the number of cells and a decrease in morphological changes can be observed compared to images only treated with CIS. NucBlue™ staining shows more stained cells in both cell lines after NIMO compared to CIS treatment alone, while fewer dead cells are visible (red, PI) (Fig. 3a). In comparison to the control, which showed a low number of dead cells of 4.0% ± 2.5% (control cells), the cell death rate increased to 54.6% ± 15.6% during 20 µM CIS treatment and decreased to 48.2% ± 14.0% after NIMO pre-treatment (20 µM NIMO) in SW10 cells (Fig. 3b). Microscopic analysis of the cell line RN33B (Fig. 3c) revealed significant alterations in morphology under CIS treatment, accompanied by a substantial decrease in cell number and cell aggregation (bright field). Pre-treatment with NIMO resulted in a further augmentation in cell count and a reduction in morphological changes when compared to images exposed exclusively to CIS. Furthermore, NucBlue™ staining revealed a higher proportion of stained cells following NIMO treatment as compared to CIS treatment alone, while the number of dead cells was reduced (red, PI) (Fig. 3c). The cell death rate of RN33B cell line increased from 0.6% ± 0.8% (control) to 83.4% ± 2.7% during CIS treatment and was reduced to 46.5% ± 6.9% by 20 µM NIMO pre-treatment (Fig. 3d). Due to the increase in cell death in the SKOV-3 cells under NIMO and CIS treatment measured with LDH assay, this has been chosen as a representative cancer cell model for the morphological analysis. A decrease in the number of cells during CIS treatment was visible, which continued to decrease with NIMO application (bright field). The total amount of CIS-treated cells stained by NucBlue™ was not significant altered in comparison to the control and cells treated with CIS and NIMO. The data indicated an elevation in the number of dead by cells treated with CIS, with a further increase observed through treatment with CIS and NIMO (PI) (Fig. 3e). While no dead cells were visible in the control, the cell death rate increased to 4.3% ± 1.6% (*p* ≤ 0.05) with CIS and to 6.0% ± 0.7% with NIMO and CIS treatment (20 µM NIMO, *p* ≤ 0.01, Fig. 3f). Further multiple statistical analysis is presented in Tables S7, S8 and S9.

### NIMO affects the activation of anti-apoptotic signaling pathways under CIS treatment

**Fig. 4:**
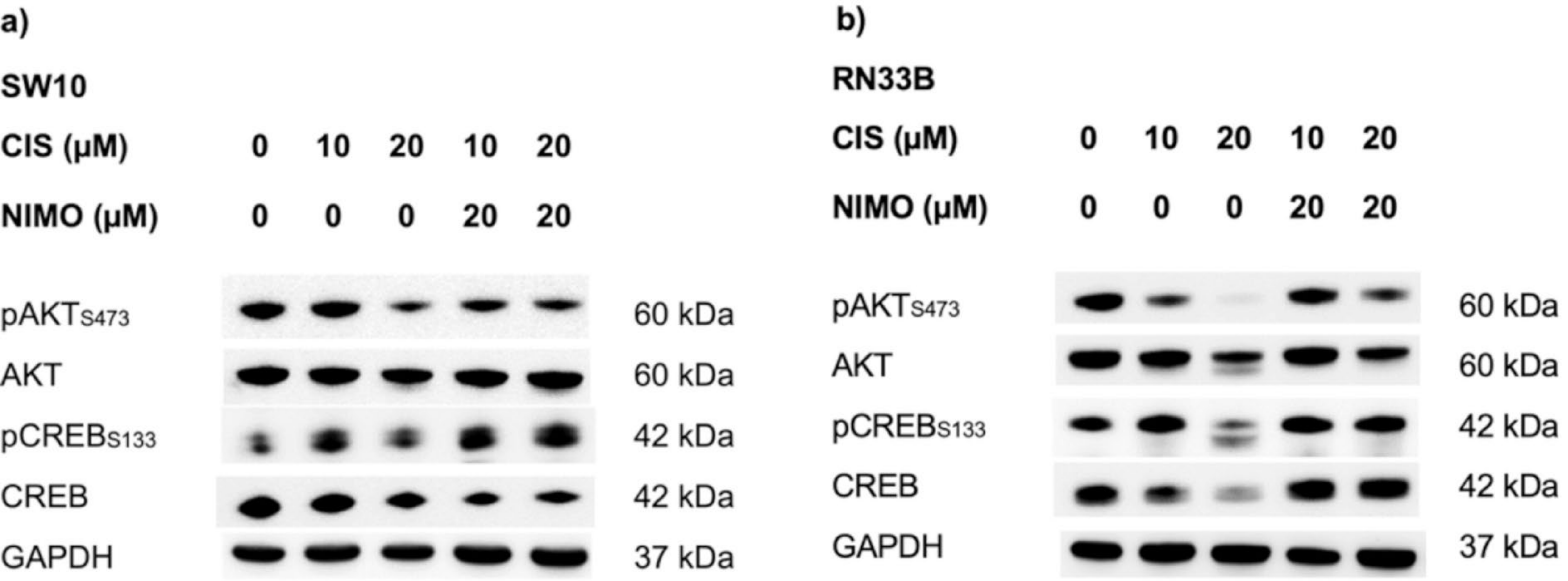
Activation of anti-apoptotic signaling pathways induced by nimodipine pre-treatment under CIS stress in Schwann cells and neuronal cells. The proteins were separated with SDS PAGE following blotting onto a nitrocellulose membrane. Afterwards specific antibodies were used to determine phosphorylation and total protein level of AKT and CREB for SW10 (**a**) and RN33B (**b**) cells. The effect of treatment was compared to the solvent control (0 µM CIS and 0 µM NIMO). GAPDH served as a loading control. The shown figures are one representative replicate out of three independent biological replicates. The full-length blots are shown in Figure S4.

Immunoblot analysis of the Schwann cells (SW10) and neuronal cells (RN33B) showed an increased activation of anti-apoptotic proteins by pre-treatment of the cells with NIMO during treatment with CIS (Fig. 4). Treatment with 10 µM CIS and 20 µM CIS leads to reduced phosphorylation of AKT at serine residue 473 in SW10 cells (Fig. 4a) and RN33B cells (Fig. 4b) compared to the control. After pre-treatment with 20 µM NIMO, there was an increase in the activation of AKT. The total protein amount of AKT remains constant and is neither influenced by the different concentrations of CIS nor by NIMO in both cell lines (Fig. 4). In addition, pre-treatment with 20 µM NIMO under CIS stress induction leads to increased phosphorylation of CREB at serine residue 133 in SW10 (Fig. 4a) and RN33B cells (Fig. 4b) after activation was reduced by CIS compared to the control. The total amount of CREB protein was not altered by NIMO and CIS treatment. GAPDH served as loading control. The graphical representation of the quantification of all three biological replicates can be found in the supplement (Figure S2, S3), as well as the corresponding multiple statistical comparisons (Table S10, S11).

**Fig. 5:**
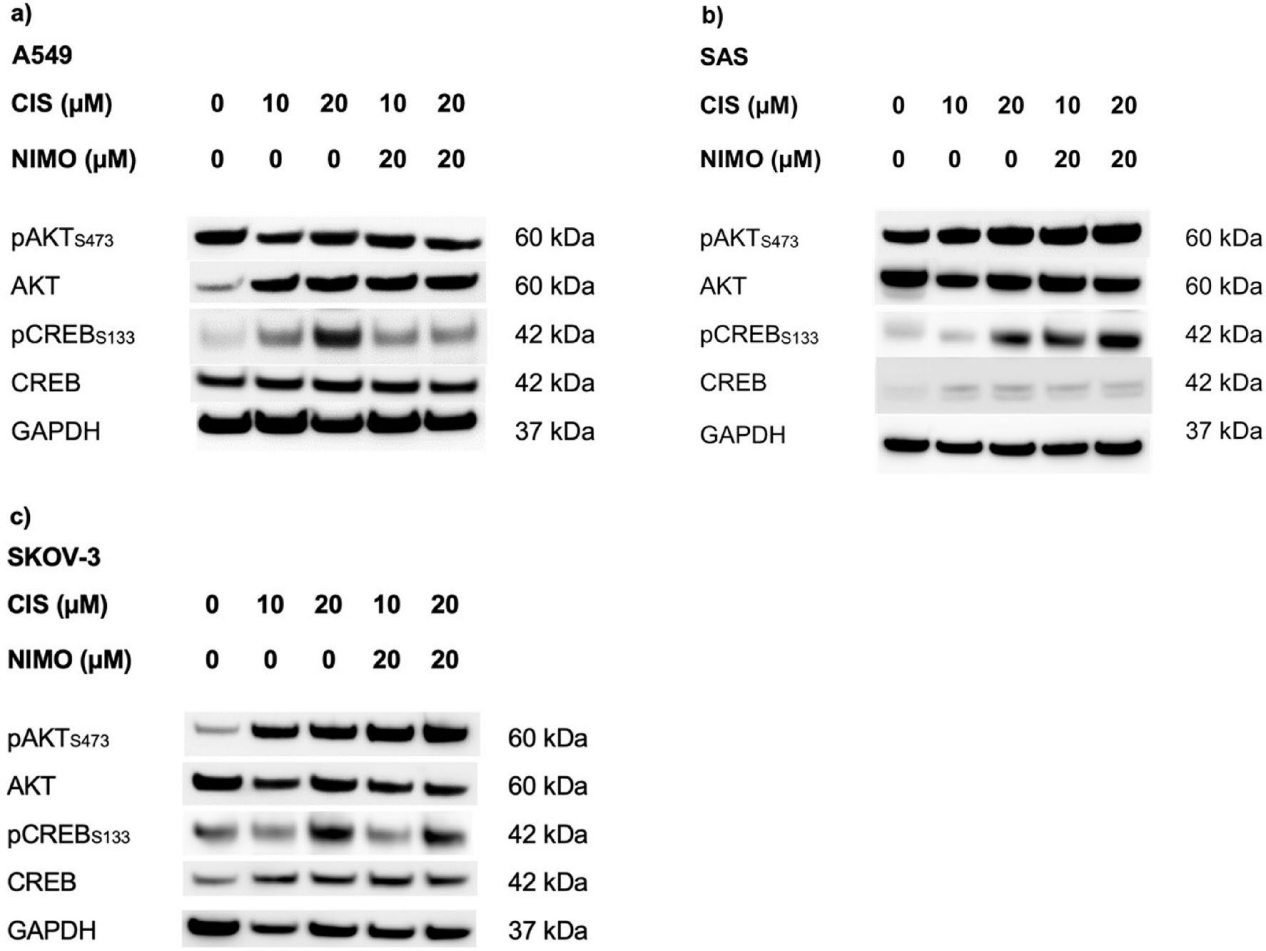
Influence of nimodipine pre-treatment on signaling pathways of various cancer cell lines during CIS application. The proteins were separated with SDS PAGE following blotting onto a nitrocellulose membrane. Afterwards specific antibodies were used to determine phosphorylation and total protein level of AKT and CREB for A549 (**a**), SAS (**b**) and SKOV-3 (**c**) cells. Treatment effect was compared to solvent control (0 µM CIS and 0 µM NIMO). GAPDH served as a loading control. The shown figures are one representative replicate out of three independent biological replicates. The full-length blots are shown in Figure S8.

With regard to the cancer cells, the treatment with 10 µM and 20 µM CIS showed no aberrant activation of AKT at the serine residue 473 in A549 (Fig. 5a), SAS (Fig. 5b) and SKOV-3 cells (Fig. 5c) compared to the control cells. Treatment with 20 µM NIMO also showed no different amount of phosphorylated AKT. The total protein amount of AKT remains unaffected by 10 µM and 20 µM CIS treatment in A549 cells (Fig. 5a) and SAS cells (Fig. 5b), while there is a slight decrease in SKOV-3 cells, which is reversed by pre-treatment with 20 µM NIMO (Fig. 5c). An increase in the phosphorylation of CREB at serine residue 133 by 10 µM and even more by 20 µM CIS is observed in all three cell lines compared to the control (Fig. 5). In the SAS cell line this effect is further enhanced by 20 µM NIMO (Fig. 5b), whereas in the A549 (Fig. 5a) and SKOV-3 (Fig. 5c) cell lines no increase or decrease in activation by NIMO is evident. There was no change in the total protein amount of CREB and AKT during CIS or NIMO treatment. GAPDH was used as a loading control. Figure S5, S6 and S7 demonstrate the quantification of all three biological replicates and the multiple statistical comparison is shown in Table S12, S13 and S14.

### NIMO upregulates the protein level of the transcription regulator LMO4 in Schwann cells, neuronal cells and various cancer cells

**Fig. 6:**
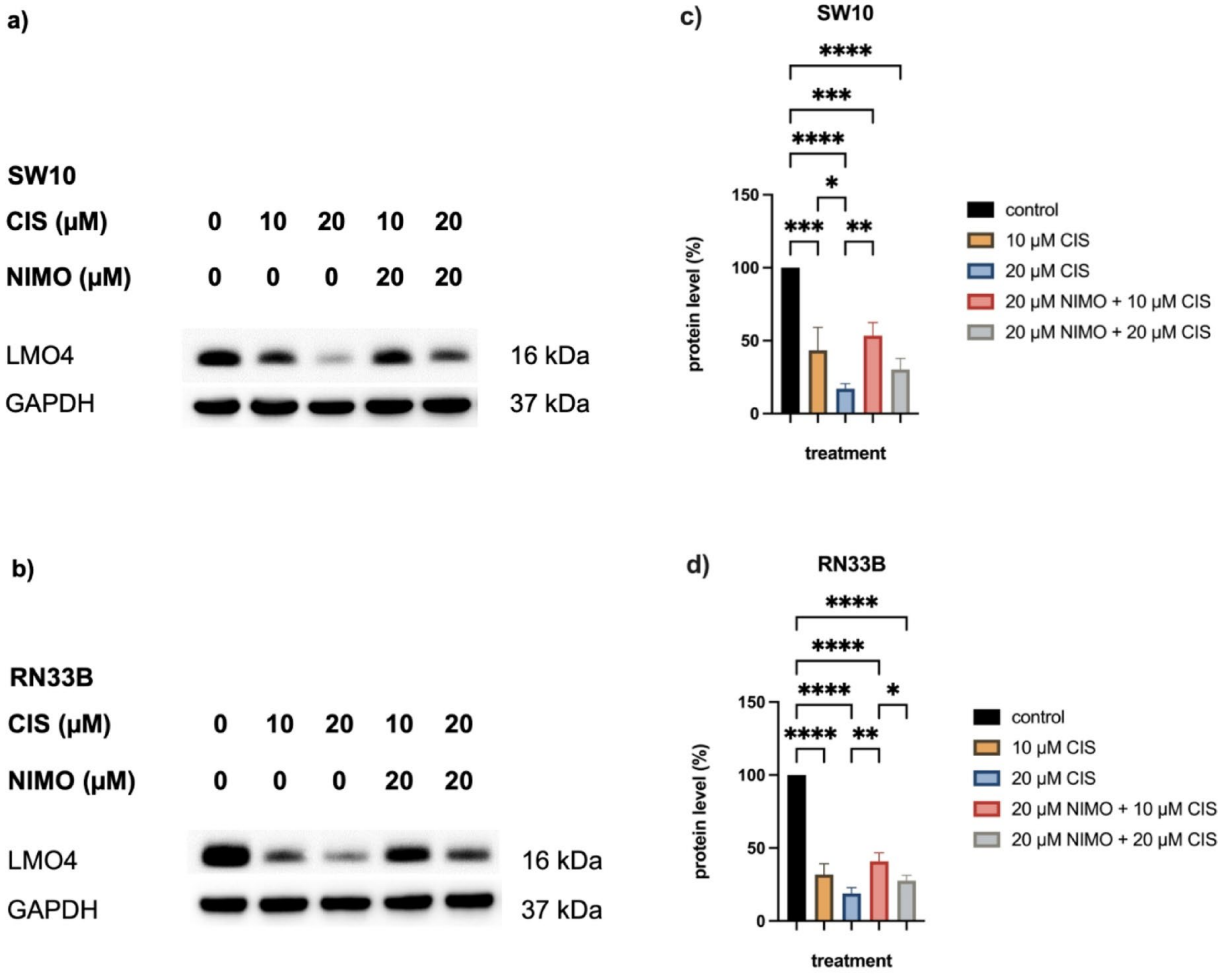
Nimodipine leads to an increase of LMO4 in Schwann cells and neuronal cells under CIS stress. The proteins were separated with SDS PAGE and then blotted onto a nitrocellulose membrane. Subsequently, total protein levels of LMO4 for SW10 (**a**) and RN33B (**b**) were determined using specific antibodies. GAPDH served as a loading control. Western blot quantification of three independent biological replicates is shown for SW10 (**c**) and RN33B (**d**). Figures show one representative immunoblot out of three independent biological replicates (**a**, **b**, **c**), while the graphs of quantification include the mean values and SD of all replicates (**d**, **e**, **f**). The full-length blots are shown in Figure S9. *p ≤ 0.05; **p ≤ 0.01; ***p ≤ 0.001; ****p ≤ 0.0001.

The Western blots showed a decrease in LMO4 total protein amount with 10 µM CIS and a further decrease after application of 20 µM CIS in SW10 (Fig. 6a) and RN33B cells (Fig. 6b). Compared to these, the protein amount of the transcriptional co-activator LMO4 increases again in cells treated with 20 µM NIMO (Fig. 6). The quantification of all three replicates confirms this effect. In SW10 cells LMO4 protein level decreases to 43.5% ± 11.0% (10 µM CIS, *p* ≤ 0.001) and to 17.1% ± 2.5% (20 µM CIS, *p* ≤ 0.0001) compared to solvent control. However, after NIMO pre-treatment only a reduction to 53.5% ± 6.4% (10 µM CIS, 20 µM NIMO, *p* ≤ 0.001) and to 30.3% ± 5.3% (20 µM CIS, 20 µM NIMO, *p* ≤ 0.0001) was visible (Fig. 6c). The neuronal cell line RN33B showed a decrease of LMO4 protein level to 31.8% ± 5.2% (10 µM CIS, *p* ≤ 0.0001) and to 18.9% ± 2.8% (20 µM CIS, *p* ≤ 0.0001) by CIS treatment, which was reduced in NIMO-treated cells to a decrease of 40.9% ± 4.1% (10 µM CIS, 20 µM NIMO, *p* ≤ 0.0001) and to 27.6% ± 2.7% (20 µM CIS, 20 µM NIMO, *p* ≤ 0.0001) compared to control cells (Fig. 6d). Additional multiple statistical comparison is shown in Tables S15 and S16.

**Fig. 7:**
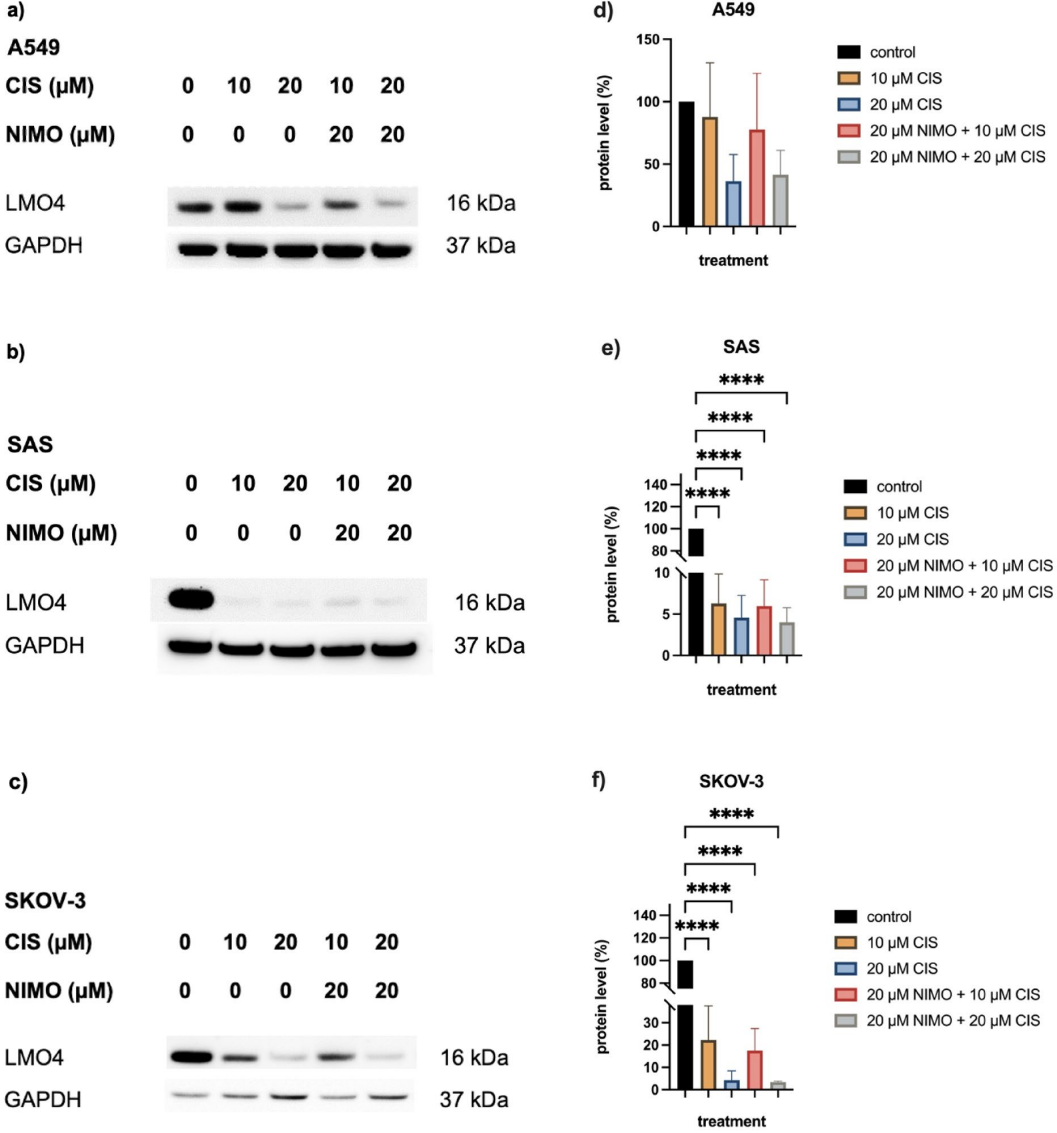
The impact of nimodipine pre-treatment on the LMO4 protein level in cancer cell lines under stress induction with CIS. The proteins were separated with SDS PAGE and then blotted onto a nitrocellulose membrane. The total protein content of LMO4 was then determined for A549 (**a**), SAS (**b**) and SKOV-3 (**c**) cells using specific antibodies. GAPDH served as a loading control. Western blot quantification of three independent biological replicates is shown for A459 (**d**), SAS (**e**) and SKOV-3 (**f**) cells. Figures show one representative immunoblot out of three independent biological replicates (**a**, **b**, **c**), while die diagrams of quantification include the mean values and SD of all replicates (**d**, **e**, **f**). GAPDH served as loading control. The full-length blots are shown in Figure S10. *p ≤ 0.05; **p ≤ 0.01; ***p ≤ 0.001; ****p ≤ 0.0001.

The immunoblot analysis of the tumor cells also showed a reduction in the amount of LMO4 protein during 10 µM CIS and 20 µM CIS application. The total amount of LMO4 remained constant in A549 cells treated with 10 µM CIS while those treated with 20 µM CIS showed a decrease (Fig. 7a). A strong decrease in the amount of LMO4 protein was observed in the SAS cells during CIS treatment (Fig. 7b). The SKOV-3 cells showed only a slight decrease compared to the control, which increased further under 20 µM CIS (Fig. 7c). Compared to the treatment with 10 µM CIS and 20 µM CIS, there was neither a significant change in the amount of LMO4 protein in all three cell lines after pre-treatment with NIMO (Fig. 7).

The quantification of all three replicates showed a decrease in the amount of LMO4 protein to 87.8% ± 30.5% (n.s.) and 36.3% ± 15.2% by CIS (n.s.) in the A549 cells compared to the control, while under NIMO treatment this also decreased to 77.7% ± 31.8% (n.s.) and 41.4% ± 13.9% (n.s., Fig. 7d). A decrease to 6.3% ± 2.5% (*p* ≤ 0.0001) and 4.6% ± 1.9% (*p* ≤ 0.0001) and in combination with NIMO pre-treatment to 6.0% ± 2.2% (*p* ≤ 0.0001) and 4.0% ± 1.2% (*p* ≤ 0.0001) was detected in the SAS cells (Fig. 7e). There was also a decrease in the protein amount of LMO4 in the SKOV-3 cells to 22.3% ± 10.3% (*p* ≤ 0.0001) and 4.3% ± 2.8% (*p* ≤ 0.0001) compared to the pre-treatment with NIMO to 17.5% ± 7.0% (*p* ≤ 0.0001) and 6.3% ± 3.6% (*p* ≤ 0.0001) compared to the control cells (Fig. 7f). Further multiple statistical analysis can be found in Table S17, S18 and S19.

## Discussion

Although NIMO was originally developed as a calcium channel antagonist for the treatment of hypertension, it has since been demonstrated to be a valuable agent for the treatment and prevention of vasospasm after aSAH^1,3^. In addition, previous studies have also shown the potential for neuroprotection and neuroregeneration^7,8,10,32,33^. In own preliminary work, we observed a protective effect on neuronal and Schwann cells under various stress conditions as well as on auditory cells^7,8^. In addition, some studies have shown a beneficial effect on the outcome of several neurodegenerative diseases^34^. The mode of action of nimodipine involves its influence on calcium influx. Given that an overload of calcium within the cell, as occurs during cisplatin therapy, can induce cell death, this suggests a potential protective effect of nimodipine^35^. Furthermore, NIMO is a well-tolerated substance. Apart from orthostatic hypotension and intolerance, no other serious adverse effects have been documented^1^. In contrast to other calcium channel inhibitors of the same class, its lipophilic structure allows it to also develop its effect in the central nervous system^2^. Previous research conducted on ex vivo models has demonstrated that the neuroprotective effect can be observed at least four hours prior to the administration of the stressor^36^. This timeframe is suitable for integration into a clinical routine.

The main limiting side effects of CIS treatment are neuro-, oto- and nephrotoxicity^17,25^ associated with the development of CIPN^20,37^, hearing loss^38,39^ and nephropathy^22^. In vitro models under CIS have demonstrated the potential for NIMO to exert a protective effect on neuronal^8^ and auditory hair cells^7^. This raises the question of whether this can also be transferred to kidney cells and thus achieve a protective effect on the kidney. The development of nephropathy as a consequence of CIS administration represents a significant obstacle to the efficacy of chemotherapy^40^. Subsequently, previous studies have demonstrated that hydration and the administration of magnesium and mannitol represent potential preventive measures^22^. Further studies are required to determine the suitability of NIMO as an adjuvant in the prevention of CIS-associated nephropathy.

The development of novel pharmaceutical agents for the treatment of chemotherapy-associated adverse effects is directed towards their impact on tumor progression. The protective effect of NIMO on cancer cells was not discernible even after 48 h. In the present study, a significant increase from 8.1% ± 2.6% to 14.3% ± 5.0% in the cytotoxic effect of CIS by NIMO was demonstrated. This observation was also shown in the study by Scheer et al. using vincristine as a stressor, where an increase of the cytotoxic effect by NIMO was demonstrated^33^. As demonstrated in prior studies, a mutation or overexpression of Cav1.2 and Cav1.3 has been identified in tumor cells^41^. These may promote tumor proliferation, migration and the development of therapy resistance. Therefore, the calcium channels are discussed as a potential target for tumor therapy^42–44^. However, further studies are needed to investigate the effect of NIMO in this mechanism in more detail.

During treatment with platinum derivates, including CIS, patients suffer from nausea and vomiting (chemotherapy-induced nausea and vomiting, CIVN)^45–47^. Cassidy et al. conducted a study at the end of the 1990s on patients with ovarian cancer in which NIMO was added to CIS therapy to reduce the rate of peripheral neuropathy during chemotherapy^48^. This was based on the evidence reducing CIPN by nimodipine. Due to poor patient compliance with oral NIMO application, triggered by CIVN, no convincing result could be achieved here. Another form of administration of NIMO should be discussed in this case, for example intravenously like in the treatment of aSAH. The combination with neurokinin (NK) -1 receptor antagonists^45^, 5-hydroxytrypamine (HT) 3 receptor antagonists^46,49^ or dexamethasone^50,51^, which are common used for treatment for CIVN^52^, is also an option to prevent poor patient compliance.

NIMO activates anti-apoptotic cell signaling pathways to exert its neuroprotective effect^7,8^. The administration of NIMO prior to the administration of CIS therapy has been demonstrated to result in the enhanced activation of AKT and CREB in neuronal and glial cells^7,8^, which in turn has been shown to reduce the rate of cell death. The activation of AKT results in the increased activation of CREB^53^, thereby reducing the apoptosis rate^54^. One hallmark of cancer cells is reaching immortality via the uncoupling of the physiological cell death processes by constitutive activation of anti-apoptotic signaling pathways. Therefore, it is not surprising that the cancer cell lines exhibit elevated levels of phosphorylated AKT to prevent apoptosis^55,56^. In comparison to neuronal and Schwann cells, NIMO does not result in further activation but rather a tendency towards reduced phosphorylation of AKT and CREB, which could consequently lead to increased apoptosis. The detailed mechanism behind this different signal pathway regulation of neuronal cells and the cancer cells in particular the role of calcium in this process, should be proved by further functional studies.

LMO4 plays a significant role in the regulation of transcription, influencing the development of the nervous system and the survival of neurons^57,58^. Furthermore, LMO4 has been demonstrated to influence the regulation of intracellular calcium concentration through the expression of receptors. This results in an additional protective effect, as maintaining homeostasis is crucial for processes such as membrane potential and cell signaling pathways^29,59^. CIS results in the downregulation of the transcriptional regulator through the initiation of oxidative stress by ROS formation, which is associated with reduced anti-apoptotic signaling pathways^25^. LMO4 activates AKT^60^ and forms transcriptional complexes with CREB^28^. NIMO pre-treatment has been observed to upregulate LMO4 in neuronal and Schwann cells, as well as in auditory sensory cells^7^ in the presence of CIS. In addition to its neuroprotective function, LIM protein 1–4 plays a role in the progression and initiation of cancer if overexpressed, and are associated with T cell leukemia, breast cancer, and neuroblastoma^26,61^. LMO4 was also initially identified as an autoantigen in breast cancer^62^ and is overexpressed in approximately half of the cases^63,64^, which is associated with a poor prognosis^61^. Our findings indicate that LMO4 levels remained constant and did not increase in the presence of NIMO, in comparison to CIS treatment alone in cancer cell lines. In the ovarian carcinoma cell line, there was a tendency for LMO4 to decrease with NIMO, which is consistent with the observed increase in cell death with the combination therapy of NIMO and CIS. This suggests that maintaining a balance of LMO4 is essential for ensuring cell survival.

The dosage of the pharmaceutical agent employed in this study was based on the findings of previous studies and is intended solely to investigate the effect and mechanism of action. It should be noted that this is not equivalent to in vivo studies, for which the optimal dosage and administration, frequency of administration and optimal timing must be determined in further studies. Furthermore, there is no evidence of a long-term effect, whether in terms of the neuroprotective impact of augmented CIS therapy or the long-term influence on cancer cells. It remains unclear whether the non-protective effect on these cells can be sustained, whether the potentiation will remain constant or even increase.

## Conclusions

Nimodipine pre-treatment has been demonstrated to exert a protective effect on both neuronal and Schwann cells under CIS therapy. The study on cell lines of non-small cell lung carcinoma, ovarian carcinoma and squamous cell carcinoma demonstrated no protective effect, but rather a tendency towards increased cell death. The neuroprotective effect was associated with an increased activation of anti-apoptotic signaling pathways and the upregulation of the transcription regulator LMO4 under CIS therapy. Therefore, NIMO pre-treatment may be a potential strategy to mitigate or even prevent CIS-associated side effects, such as neuro- and ototoxicity, while maintaining the desired cytotoxic effect. This has the potential to facilitate not only the optimization of chemotherapy and its efficacy, but also a notable enhancement in the quality of life for patients undergoing chemotherapy.

## Electronic supplementary material

Below is the link to the electronic supplementary material.

Supplementary Material 1

## Data Availability

Data is provided within the manuscript or supplementary information files. Further inquiries can be directed to the corresponding author.
