# Supplementary material for: Nimodipine protects Schwann and neuronal cells from cell death induced by cisplatin without affecting cancer cells
Source: Sci Rep. 2025 Jun 25;15:20279. doi: 10.1038/s41598-025-06854-5 (PMC12198361; doi:10.1038/s41598-025-06854-5)
Supplement: Supplementary file 1 — Supplementary Material 1 [file 41598_2025_6854_MOESM1_ESM.pdf]

Supplementary Figures

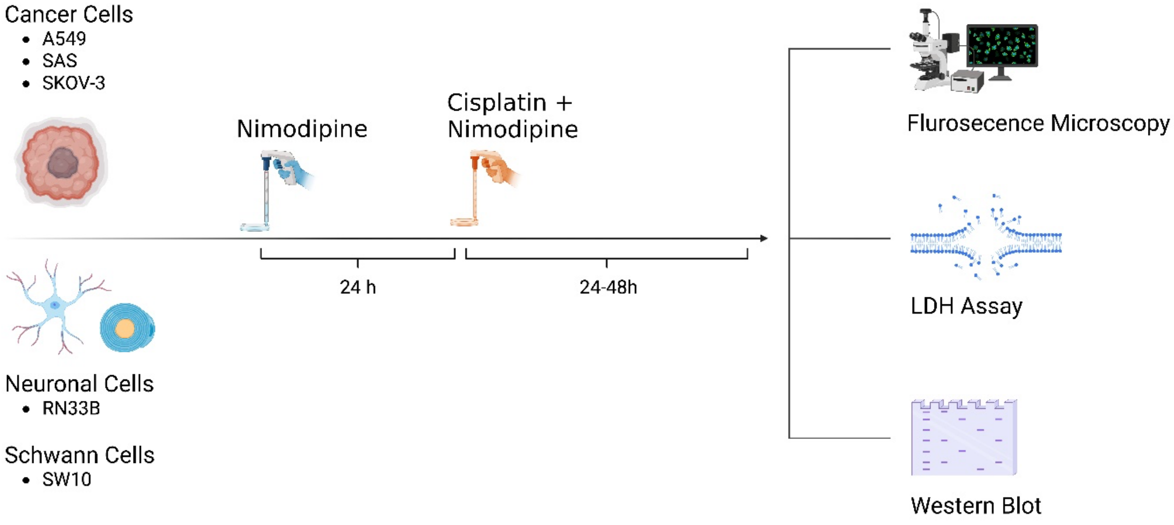

Figure S1: Overview of treatment scheme and experimental set-up (Created in BioRender®. Agreement number: CQ288BCBE9, Scheer, M. (2025) https://BioRender.com/onqp31v)

Table S1: Antibodies used for immunoblotting

Antibody	Species	Protein loading on SDS PAGE	Dilution	Dilution buffer	Manufacture
AKT (40D4) #2920	Mouse IgG1	30 µg proteins	1:2000	5 % MP in TBS-T	Cell Signaling Technology (Danvers, MA, USA)
Phospho-Akt (Ser473) (D9E) #4060	Rabbit IgG	30 µg proteins	1:1000	5 % BSA in TBS-T	Cell Signaling Technology (Danvers, MA, USA)
CREB (48H2) #9197	Rabbit IgG	30 µg proteins	1:1000	5 % BSA in TBS-T	Cell Signaling Technology (Danvers, MA, USA)
Phospho-CREB (Ser133) (87G3) #9198	Rabbit IgG	30 µg proteins	1:1000	5 % MP in TBS-T	Cell Signaling Technology (Danvers, MA, USA)
LMO4 (D6V4Z) #81428	Rabbit IgG	50 µg proteins	1:1000	5 % BSA in TBS-T	Cell Signaling Technology (Danvers, MA, USA)
GAPDH (14C10) #2118	Rabbit IgG	30 µg proteins	1:1000	5 % BSA in TBS-T	Cell Signaling Technology (Danvers, MA, USA)
Anit-Rabbit IgG. HRP-linked Antibody #7074	Goat		1:1000	2 % MP in TBS-T	Cell Signaling Technologie Inc. (Danvers. MA. USA)
Anti-Mouse IgG. HRP-linked Antibody #7076	Horse		1:1000	2 % MP in TBS-T	Cell Signaling Technologie Inc. (Danvers. MA. USA)

Abbreviations: AKT, Protein kinase B; BSA, bovine serum albumin; CREB, cAMP response element-binding protein; GAPDH, glyceraldehyde-3-phosphatedehydrogenase; HRP, horseradish peroxidase; IgG, immunoglobulin G; LMO4, LIM domain only; MP, milk powder; TBS-T, tris-bufferd saline with 0.1 % Tween 20

Table S2. LDH assay statistics and confidence intervals (CI) of SW10 cells

**SW10 cells**	*Cell death [%] Mean Diff.*	*95.00 % CI of diff.*	*summary*	*adjusted p value*
without stress				
control vs. 10 µM NIMO	0.5545	-18.02 to 19.13	ns	0.9965
control vs. 20 µM NIMO	-0.3787	-18.96 to 18.20	ns	0.9984
10 µM NIMO vs. 20 µM NIMO	-0.9333	-19.51 to 17.65	ns	0.9902
20 µM CIS (24h)				
control vs. 10 µM NIMO	13.62	-4.956 to 32.20	ns	0.1657
control vs. 20 µM NIMO	22.24	3.657 to 40.81	*	0.0196
10 µM NIMO vs. 20 µM NIMO	8.613	-9.966 to 27.19	ns	0.4553
20 µM CIS (48h)				
control vs. 10 µM NIMO	11.39	-6.667 to 29,46	ns	0.2671
control vs. 20 µM NIMO	18.77	0.7048 to 36,83	*	0.0410
10 µM NIMO vs. 20 µM NIMO	7.372	-10.69 to 25.43	ns	0.5611

Diff.: Difference; * $p<0.05$; control: absolute ethanol; NIMO: nimodipine; CIS: cisplatin; ns: not significant

Table S3. LDH assay statistics and CI of RN33B cells

**RN33B cells**	*Cell death [%] Mean Diff.*	*95.00 % CI of diff.*	*summary*	*adjusted p value*
without stress				
control vs. 10 µM NIMO	0.3603	-4.734 to 5.455	ns	0.9806
control vs. 20 µM NIMO	-0.01593	-5.110 to 5.079	ns	>0.9999
10 µM NIMO vs. 20 µM NIMO	-0.3762	-5.471 to 4.718	ns	0.9789
20 µM CIS (24h)				
control vs. 10 µM NIMO	5.685	0.5901 to 10.78	*	0.0289
control vs. 20 µM NIMO	15.60	10.50 to 20.69	****	<0.0001
10 µM NIMO vs. 20 µM NIMO	9.914	4.820 to 15.01	***	0.0006
20 µM CIS (48h)				
control vs. 10 µM NIMO	8.589	2.185 to 14.99	**	0.0081
control vs. 20 µM NIMO	14.83	8.428 to 21.24	****	<0.0001
10 µM NIMO vs. 20 µM NIMO	6.243	-0.1603 to 12.65	ns	0.0567

Diff.: Difference; * $p \leq 0.05$; ** $p \leq 0.01$; *** $p \leq 0.001$; **** $p \leq 0.0001$; control: absolute ethanol; NIMO: nimodipine; CIS: cisplatin; ns: not significant

Table S4. LDH assay statistics and CI of A549 cells

**A549 cells**	*Cell death [%] Mean Diff.*	*95.00 % CI of diff.*	*summary*	*adjusted p value*
without stress				
control vs. 10 µM NIMO	-0.7220	-5.385 to 3.941	ns	0.9152
control vs. 20 µM NIMO	-3.117	-7.781 to 1.546	ns	0.2245
10 µM NIMO vs. 20 µM NIMO	-2.395	-7.059 to 2.268	ns	0.3988
20 µM CIS (24h)				
control vs. 10 µM NIMO	-0.5495	-5.213 to 4.114	ns	0.9498
control vs. 20 µM NIMO	-1.697	-6.360 to 2.966	ns	0.6211
10 µM NIMO vs. 20 µM NIMO	-1.148	-5.811 to 3.516	ns	0.8011
20 µM CIS (48h)				
control vs. 10 µM NIMO	-0.4632	-6.175 to 5.248	ns	0.9759
control vs. 20 µM NIMO	-1.261	-6.973 to 4.450	ns	0.8361
10 µM NIMO vs. 20 µM NIMO	-0.7979	-6.509 to 4.914	ns	0.9303

Diff.: Difference; control: absolute ethanol; NIMO: nimodipine; CIS: cisplatin; ns: not significant

Table S5. LDH assay statistics and CI of SAS cells

**SAS cells**	*Cell death [%] Mean Diff.*	*95.00 % CI of diff.*	*summary*	*adjusted p value*
without stress				
control vs. 10 µM NIMO	-2.204	-12.43 to 8.022	ns	0.8477
control vs. 20 µM NIMO	-2.820	-13.05 to 7.406	ns	0.7643
10 µM NIMO vs. 20 µM NIMO	-0.6159	-10.84 to 9.611	ns	0.9871
20 µM CIS (24h)				
control vs. 10 µM NIMO	0.2305	-9.996 to 10.46	ns	0.9982
control vs. 20 µM NIMO	-0.5049	-10.73 to 9.722	ns	0.9913
10 µM NIMO vs. 20 µM NIMO	-0.7354	-10.96 to 9.491	ns	0.9816
20 µM CIS (48h)				
control vs. 10 µM NIMO	4.178	-6.048 to 14.40	ns	0.5605
control vs. 20 µM NIMO	3.293	-6.933 to 13.52	ns	0.6946
10 µM NIMO vs. 20 µM NIMO	-0.8846	-11.11 to 9.342	ns	0.9735

Diff.: Difference; control: absolute ethanol; NIMO: nimodipine; CIS: cisplatin; ns: not significant

Table S6. LDH assay statistics and CI of SKOV-3 cells

**SKOV-3 cells**	*Cell death [%] Mean Diff.*	*95.00 % CI of diff.*	*summary*	*adjusted p value*
without stress				
control vs. 10 µM NIMO	-0.9196	-6.598 to 4.759	ns	0.9106
control vs. 20 µM NIMO	-2.897	-8.575 to 2.782	ns	0.4123
10 µM NIMO vs. 20 µM NIMO	-1.977	-7.656 to 3.702	ns	0.6542
20 µM CIS (24h)				
control vs. 10 µM NIMO	-0.5934	-6.272 to 5.085	ns	0.9616
control vs. 20 µM NIMO	-1.797	-7.476 to 3.882	ns	0.7032
10 µM NIMO vs. 20 µM NIMO	-1.204	-6.883 to 4.475	ns	0.8523
20 µM CIS (48h)				
control vs. 10 µM NIMO	-2.370	-8.049 to 3.309	ns	0.5470
control vs. 20 µM NIMO	-6.194	-11.87 to -0.5154	*	0.0313
10 µM NIMO vs. 20 µM NIMO	-3.824	-9.503 to 1.855	ns	0.2256

Diff.: Difference; * p ≤ 0.05; control: absolute ethanol; NIMO: nimodipine; CIS: cisplatin; ns: not significant

Table S7. Immunofluorescence quantification statistics and CI of SW10 cells

**SW10 cells**	*Cell death [%] Mean Diff.*	*95.00 % CI of diff.*	*summary*	*adjusted p value*
control vs. 20 µM CIS	-50.58	-93.77 to -7.396	*	0.0266
control vs. 20 µM CIS + 20 µM NIMO	-44.20	-87.39 to -1.016	*	0.0458
20 µM CIS vs. 20 µM CIS + 20 µM NIMO	6.380	-36.81 to 49.57	ns	0.8948

Diff.: Difference; * p ≤ 0.05; control: solvent control (EtOH, NaCl); NIMO: nimodipine; CIS: cisplatin; ns: not significant

Table S8. Immunofluorescence quantification statistics and CI of RN33B cells

**RN33B cells**	*Cell death [%] Mean Diff.*	*95.00 % CI of diff.*	*summary*	*adjusted p value*
control vs. 20 µM CIS	-82.76	-103.7 to -61.79	***	0.0003
control vs. 20 µM CIS + 20 µM NIMO	-45.87	-66.84 to -24.90	**	0.0032
20 µM CIS vs. 20 µM CIS + 20 µM NIMO	36.89	13.92 to 59.87	*	0.0101

Diff.: Difference; * p ≤ 0.05; ** p ≤ 0.01; *** p ≤ 0.001; control: solvent control (EtOH, NaCl); NIMO: nimodipine; CIS: cisplatin; ns: not significant

Table S9. Immunofluorescence quantification statistics and CI of SKOV-3 cells

**SKOV-3 cells**	*Cell death [%] Mean Diff.*	*95.00 % CI of diff.*	*summary*	*adjusted p value*
control vs. 20 µM CIS	-4.340	-7.985 to -0.6944	*	0.0248
control vs. 20 µM CIS + 20 µM NIMO	-5.988	-9.633 to -2.343	**	0.0056
20 µM CIS vs. 20 µM CIS + 20 µM NIMO	-1.649	-5.294 to 1.996	ns	0.4041

Diff.: Difference; * p ≤ 0.05; ** p ≤ 0.01; control: solvent control (EtOH, NaCl) NIMO: nimodipine; CIS: cisplatin; ns: not significant

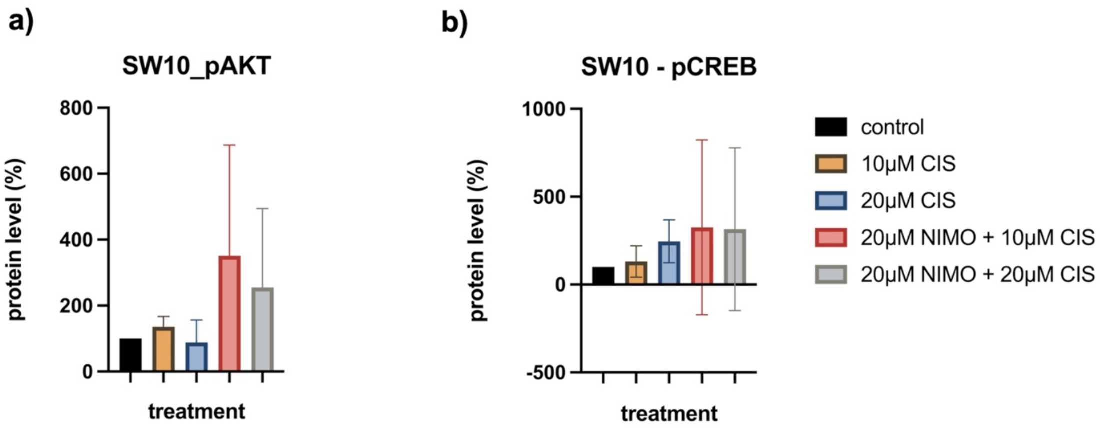

Figure S2: Quantification of SW10 Western Blots.

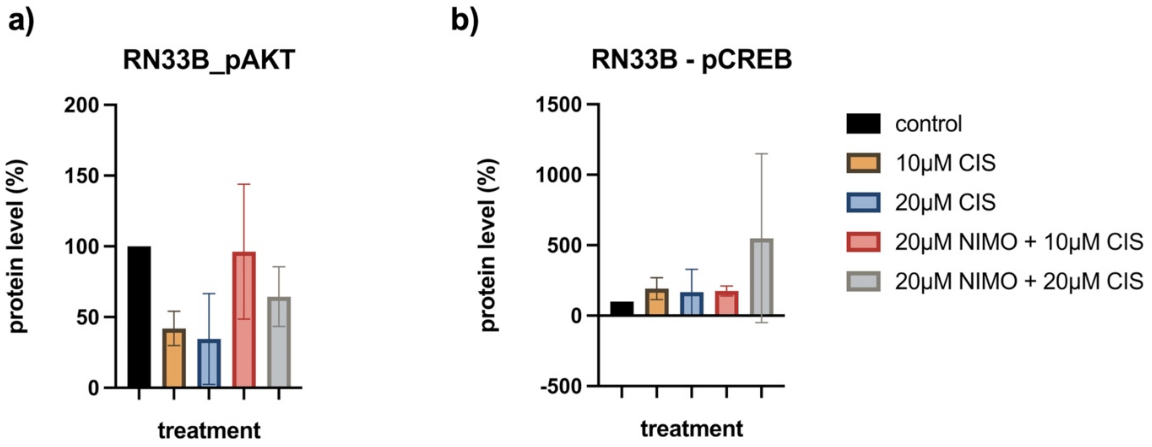

Figure S3: Quantification of RN33B Western Blots.

Table S10. pAKT and pCREB amount quantification statistical analysis and CI of SW10 cells

**SW10 cells**	*Protein level [%] Mean Diff.*	*95.00% CI of diff.*	*summary*	*adjusted p value*
pAKT				
control vs. 10 µM CIS	-35.79	-539.1 to 467.5	ns	0.9992
control vs. 20 µM CIS	11.44	-491.9 to 514.8	ns	>0.9999
control vs. 20 µM NIMO + 10 µM CIS	-251.2	-754.5 to 252.2	ns	0.5059

control vs. 20 µM NIMO + 20 µM CIS	-155.3	-658.7 to 348.0	ns	0.8427
10 µM CIS vs. 20 µM CIS	47.23	-456.1 to 550.6	ns	0.9977
10 µM CIS vs. 20 µM NIMO + 10 µM CIS	-215.4	-718.7 to 288.0	ns	0.6365
10 µM CIS vs. 20 µM NIMO + 20 µM CIS	-119.5	-622.9 to 383.8	ns	0.9303
20 µM CIS vs. 20 µM NIMO + 10 µM CIS	-262.6	-765.9 to 240.7	ns	0.4662
20 µM CIS vs. 20 µM NIMO + 20 µM CIS	-166.8	-670.1 to 336.6	ns	0.8077
20 µM NIMO + 10 µM CIS vs. 20 µM NIMO + 20 µM CIS	95.83	-407.5 to 599.2	ns	0.9672
pCREB				
control vs. 10 µM CIS	-31.33	-868.0 to 805.4	ns	>0.9999
control vs. 20 µM CIS	-146.2	-982.9 to 690.5	ns	0.9759
control vs. 20 µM NIMO + 10 µM CIS	-225.4	-1062 to 611.3	ns	0.8956
control vs. 20 µM NIMO + 20 µM CIS	-215.2	-1052 to 621.5	ns	0.9097
10 µM CIS vs. 20 µM CIS	-114.9	-951.6 to 721.8	ns	0.9900
10 µM CIS vs. 20 µM NIMO + 10 µM CIS	-194.0	-1031 to 642.7	ns	0.9356
10 µM CIS vs. 20 µM NIMO + 20 µM CIS	-183.9	-1021 to 652.8	ns	0.9462
20 µM CIS vs. 20 µM NIMO + 10 µM CIS	-79.15	-915.9 to 757.6	ns	0.9976
20 µM CIS vs. 20 µM NIMO + 20 µM CIS	-68.97	-905.7 to 767.7	ns	0.9986
20 µM NIMO + 10 µM CIS vs. 20 µM NIMO + 20 µM CIS	10.18	-826.5 to 846.9	ns	>0.9999

Diff.: Difference; control: solvent control (absolute ethanol, NaCl); NIMO: nimodipine; CIS: cisplatin; ns: not significant

Table S11. pAKT and pCREB amount quantification statistical analysis and CI of RN33B cells

**RN33B cells**	*Mean Diff.*	*95.00% CI of diff.*	*summary*	*adjusted p value*
pAKT				

control vs.<br>10 µM CIS	58.02	-16.92 to 133.0	ns	0.1552
control vs.<br>20 µM CIS	65.48	-9.466 to 140.4	ns	0.0949
control vs.<br>20 µM NIMO + 10 µM CIS	3.759	-71.18 to 78.70	ns	0.9998
control vs.<br>20 µM NIMO + 20 µM CIS	35.50	-39.44 to 110.4	ns	0.5515
10 µM CIS vs.<br>20 µM CIS	7.459	-67.48 to 82.40	ns	0.9971
10 µM CIS vs.<br>20 µM NIMO + 10 µM CIS	-54.26	-129.2 to 20.68	ns	0.1971
10 µM CIS vs.<br>20 µM NIMO + 20 µM CIS	-22.51	-97.45 to 52.43	ns	0.8546
20 µM CIS vs.<br>20 µM NIMO + 10 µM CIS	-61.72	-136.7 to 13.22	ns	0.1219
20 µM CIS vs.<br>20 µM NIMO + 20 µM CIS	-29.97	-104.9 to 44.97	ns	0.6882
20 µM NIMO +<br>10 µM CIS vs.<br>20 µM NIMO +<br>20 µM CIS	31.74	-43.20 to 106.7	ns	0.6445
pCREB				
control vs.<br>10 µM CIS	-92.09	-1217 to 1032	ns	0.9966
control vs.<br>20 µM CIS	-68.08	-1193 to 1056	ns	0.9989
control vs.<br>20 µM NIMO + 10 µM CIS	-75.22	-1200 to 1049	ns	0.9985
control vs.<br>20 µM NIMO + 20 µM CIS	-450.0	-1574 to 674.5	ns	0.5509
10 µM CIS vs.<br>20 µM CIS	24.02	-1100 to 1149	ns	>0.9999
10 µM CIS vs.<br>20 µM NIMO + 10 µM CIS	16.87	-1108 to 1141	ns	>0.9999
10 µM CIS vs.<br>20 µM NIMO + 20 µM CIS	-357.9	-1482 to 766.6	ns	0.7153
20 µM CIS vs.<br>20 µM NIMO + 10 µM CIS	-7.146	-1132 to 1117	ns	>0.9999
20 µM CIS vs.<br>20 µM NIMO + 20 µM CIS	-381.9	-1506 to 742.6	ns	0.6720
20 µM NIMO +<br>10 µM CIS vs.<br>20 µM NIMO +<br>20 µM CIS	-374.8	-1499 to 749.7	ns	0.6849

Diff.: Difference; control: solvent control (absolute ethanol, NaCl); NIMO: nimodipine; CIS: cisplatin; ns: not significant

a)

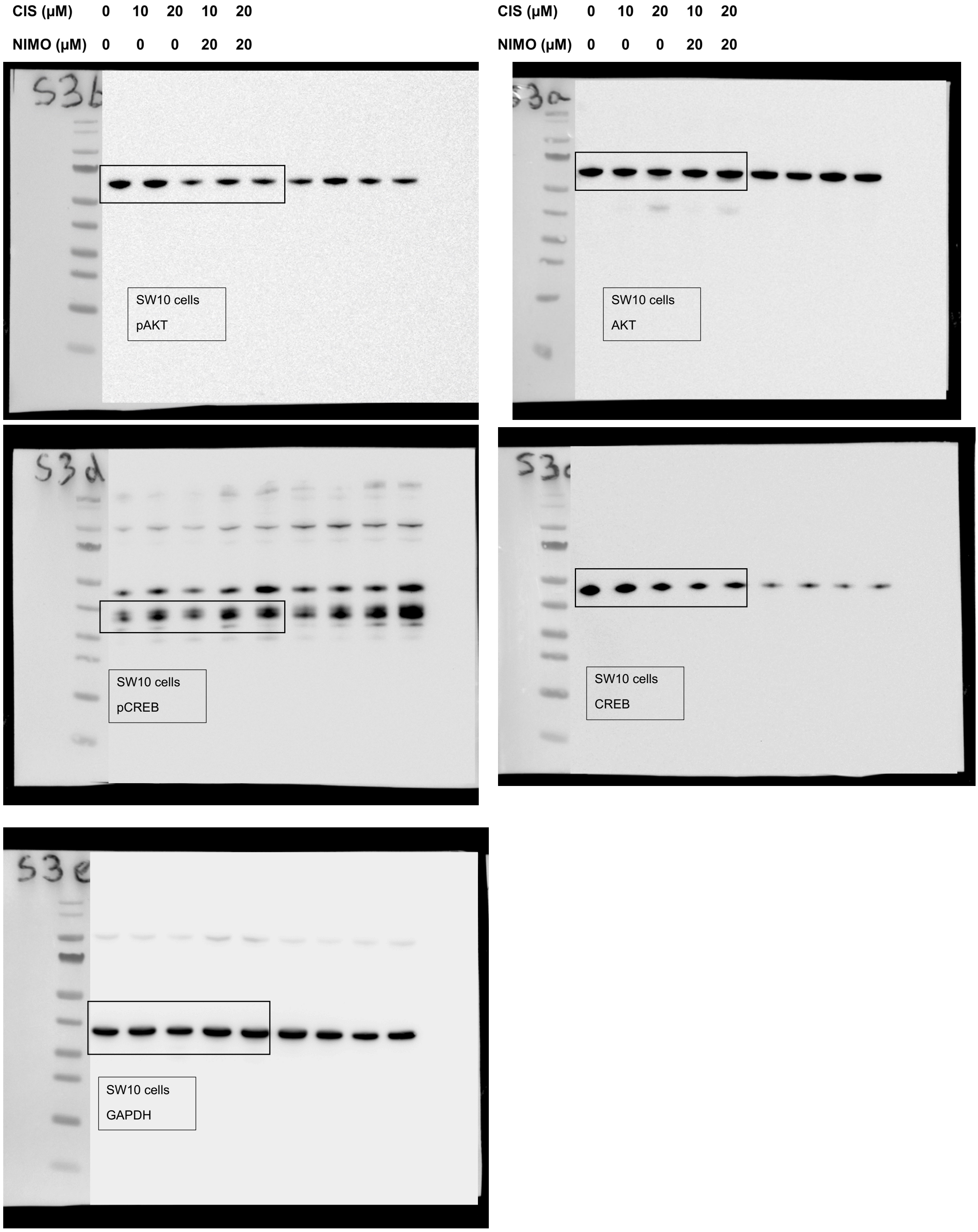
CIS (µM) 0 10 20 10 20
NIMO (µM) 0 0 0 20 20
CIS (µM) 0 10 20 10 20
NIMO (µM) 0 0 0 20 20
S3b
SW10 cells
pAKT
S3a
SW10 cells
AKT
S3d
SW10 cells
pCREB
S3c
SW10 cells
CREB
S3e
SW10 cells
GAPDH

b)

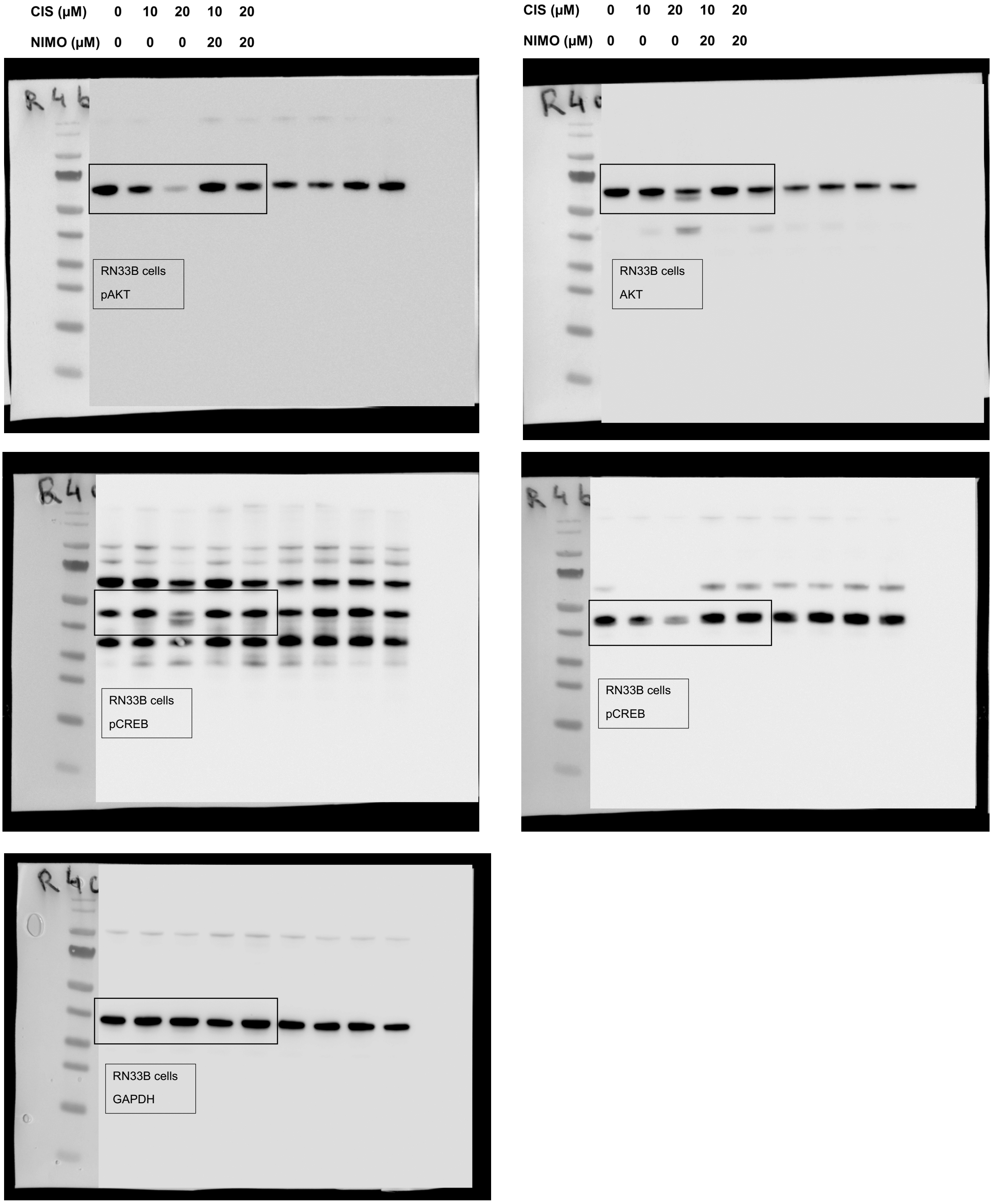

Figure S4: Full-length Western blot membranes used to prepare Figure 4. The immunoblots were prepared as described in the material and methods section. The antibody source and dilutions used are listed in Table S1. PageRuler (180-10 kDa, #26616, Thermo Fisher Scientific, Waltham, MA, USA) was used as molecular weight marker. Shown are the blots for SW10 cells (a) and RN33B cells (b).

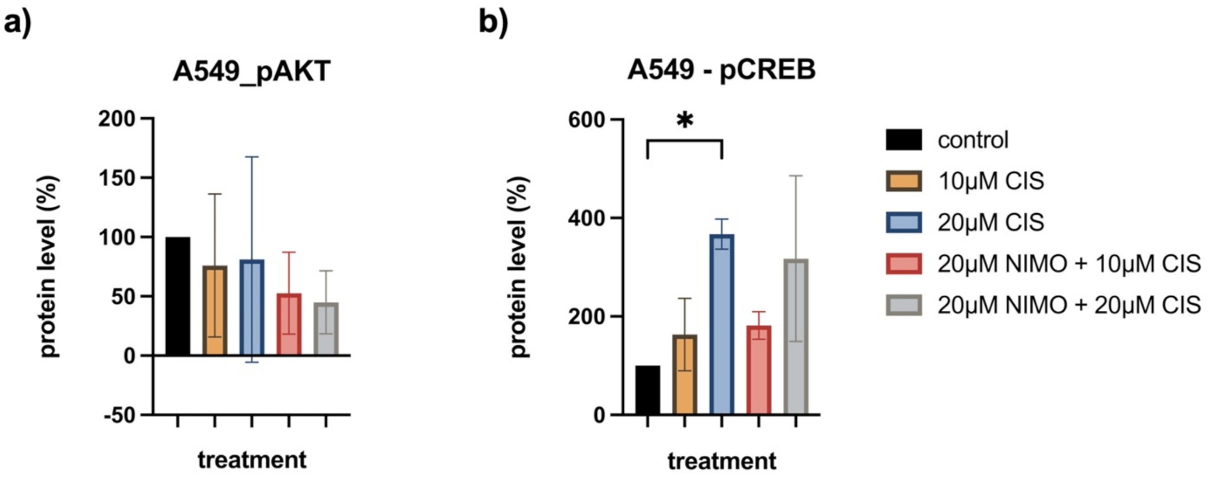

Figure S5: Graphical illustration of the quantification of A549 Western blots.

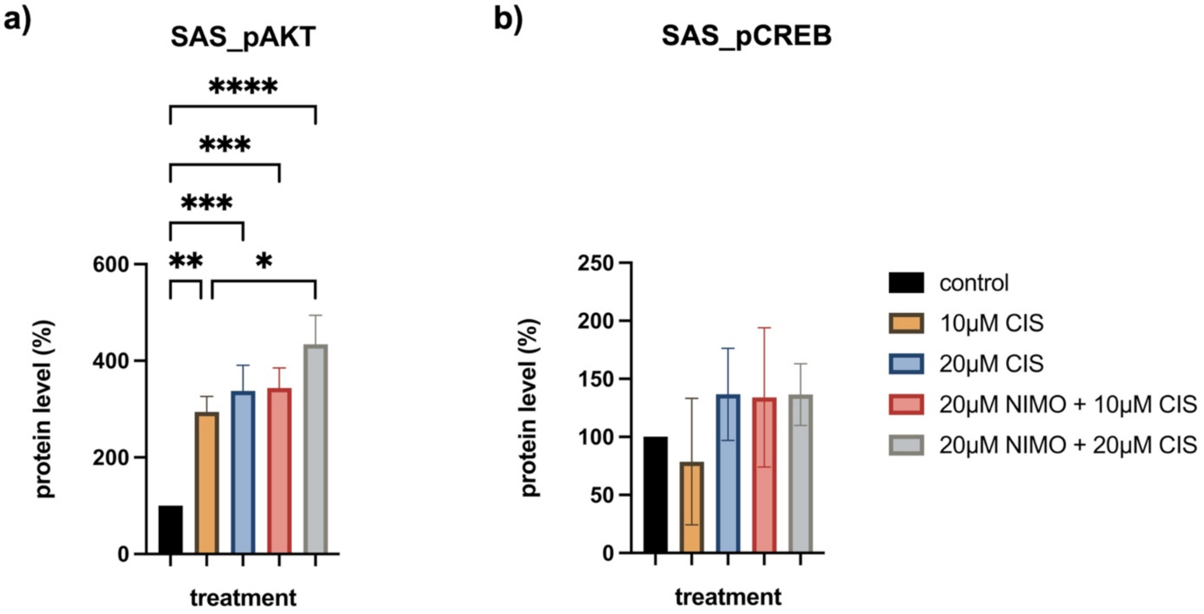

Figure S6: Graphical illustration of the quantification of SAS Western blots.

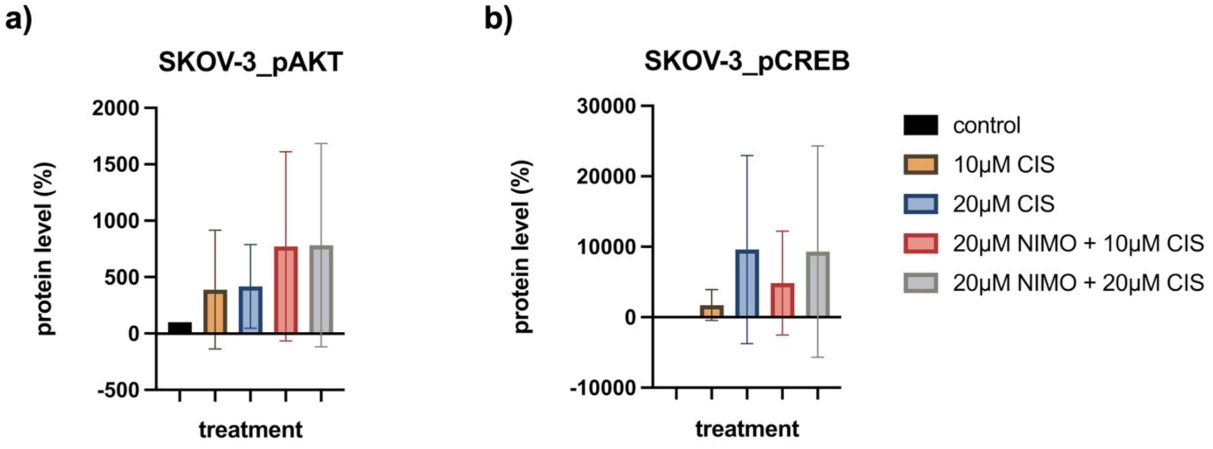

Figure S7: Graphical illustration of the quantification of SKOV-3 Western Blots.

Table S12. pAKT and pCREB amount quantification statistical analysis and CI of A549 cells

**A549 cells**	*Protein level [%] Mean Diff.*	*95.00 % CI of diff.*	*summary*	*adjusted p value*
pAKT				
control vs. 10 µM CIS	24.03	-113.1 to 161.1	ns	0.9756
control vs. 20 µM CIS	18.88	-118.2 to 156.0	ns	0.9899
control vs. 20 µM NIMO + 10 µM CIS	47.31	-89.79 to 184.4	ns	0.7852
control vs. 20 µM NIMO + 20 µM CIS	55.04	-82.06 to 192.1	ns	0.6855
10 µM CIS vs. 20 µM CIS	-5.154	-142.3 to 131.9	ns	>0.9999
10 µM CIS vs. 20 µM NIMO + 10 µM CIS	23.27	-113.8 to 160.4	ns	0.9783
10 µM CIS vs. 20 µM NIMO + 20 µM CIS	31.00	-106.1 to 168.1	ns	0.9408
20 µM CIS vs. 20 µM NIMO + 10 µM CIS	28.43	-108.7 to 165.5	ns	0.9559
20 µM CIS vs. 20 µM NIMO + 20 µM CIS	36.16	-100.9 to 173.3	ns	0.9023
20 µM NIMO + 10 µM CIS vs. 20 µM NIMO + 20 µM CIS	7.733	-129.4 to 144.8	ns	0.9997
pCREB				
control vs. 10 µM CIS	-63.25	-289.6 to 163.1	ns	0.8829
control vs. 20 µM CIS	-267.4	-493.7 to -41.09	*	0.0198
control vs. 20 µM NIMO + 10 µM CIS	-81.69	-308.0 to 144.6	ns	0.7581
control vs. 20 µM NIMO + 20 µM CIS	-217.4	-443.7 to 8.876	ns	0.0611
10 µM CIS vs. 20 µM CIS	-204.1	-430.5 to 22.16	ns	0.0823
10 µM CIS vs. 20 µM NIMO + 10 µM CIS	-18.44	-244.7 to 207.9	ns	0.9987
10 µM CIS vs. 20 µM NIMO + 20 µM CIS	-154.2	-380.5 to 72.13	ns	0.2401
20 µM CIS vs. 20 µM NIMO + 10 µM CIS	185.7	-40.60 to 412.0	ns	0.1236
20 µM CIS vs. 20 µM NIMO + 20 µM CIS	49.97	-176.3 to 276.3	ns	0.9453

20 µM NIMO + 10 µM CIS vs. 20 µM NIMO + 20 µM CIS	-135.7	-362.1 to 90.57	ns	0.3428

Diff.: Difference; * p ≤ 0.05; control: solvent control (absolute ethanol, 0.9% NaCl); NIMO: nimodipine; CIS: cisplatin; ns: not significant

Table S13. pAKT and pCREB amount quantification statistical analysis and CI of SAS cells

**SAS**	*Protein level [%] Mean Diff.*	*95.00% CI of diff.*	*summary*	*adjusted p value*
pAKT				
control vs. 10 µM CIS	-194.1	-308.8 to -79.37	**	0.0017
control vs. 20 µM CIS	-238.0	-352.7 to -123.3	***	0.0003
control vs. 20 µM NIMO + 10 µM CIS	-244.0	-358.7 to -129.2	***	0.0003
control vs. 20 µM NIMO + 20 µM CIS	-334.4	-449.1 to -219.7	****	<0.0001
10 µM CIS vs. 20 µM CIS	-43.89	-158.6 to 70.82	ns	0.7197
10 µM CIS vs. 20 µM NIMO + 10 µM CIS	-49.87	-164.6 to 64.84	ns	0.6237
10 µM CIS vs. 20 µM NIMO + 20 µM CIS	-140.3	-255.0 to -25.57	*	0.0161
20 µM CIS vs. 20 µM NIMO + 10 µM CIS	-5.975	-120.7 to 108.7	ns	0.9998
20 µM CIS vs. 20 µM NIMO + 20 µM CIS	-96.38	-211.1 to 18.33	ns	0.1122
20 µM NIMO + 10 µM CIS vs. 20 µM NIMO + 20 µM CIS	-90.41	-205.1 to 24.30	ns	0.1450
pCREB				
control vs. 10 µM CIS	21.25	-91.77 to 134.3	ns	0.9687
control vs. 20 µM CIS	-36.68	-149.7 to 76.34	ns	0.8184
control vs. 20 µM NIMO + 10 µM CIS	-34.04	-147.1 to 78.99	ns	0.8535
control vs. 20 µM NIMO + 20 µM CIS	-36.51	-149.5 to 76.52	ns	0.8208
10 µM CIS vs. 20 µM CIS	-57.93	-171.0 to 55.09	ns	0.4819
10 µM CIS vs. 20 µM NIMO + 10 µM CIS	-55.29	-168.3 to 57.74	ns	0.5235
10 µM CIS vs.	-57.76	-170.8 to 55.26	ns	0.4846

20 µM NIMO + 20 µM CIS				
20 µM CIS vs. 20 µM NIMO + 10 µM CIS	2.646	-110.4 to 115.7	ns	>0.9999
20 µM CIS vs. 20 µM NIMO + 20 µM CIS	0.1719	-112.9 to 113.2	ns	>0.9999
20 µM NIMO + 10 µM CIS vs. 20 µM NIMO + 20 µM CIS	-2.474	-115.5 to 110.6	ns	>0.9999

Diff.: Difference; * p ≤ 0.05; ** p ≤ 0.01; *** p ≤ 0.001; **** p ≤ 0.0001; control: solvent control (absolute ethanol, 0.9% NaCl); NIMO: nimodipine; CIS: cisplatin; ns: not significant

Table S14: pAKT and pCREB amount quantification statistical analysis and CI of SKOV-3 cells

**SKOV-3 cells**	*Protein level [%] Mean Diff.*	*95.00% CI of diff.*	*summary*	*adjusted p value*
pAKT				
control vs. 10 µM CIS	-289.0	-1958 to 1380	ns	0.9767
control vs. 20 µM CIS	-317.7	-1987 to 1352	ns	0.9673
control vs. 20 µM NIMO + 10 µM CIS	-672.4	-2342 to 997.0	ns	0.6831
control vs. 20 µM NIMO + 20 µM CIS	-683.2	-2353 to 986.2	ns	0.6711
10 µM CIS vs. 20 µM CIS	-28.68	-1698 to 1641	ns	>0.9999
10 µM CIS vs. 20 µM NIMO + 10 µM CIS	-383.4	-2053 to 1286	ns	0.9376
10 µM CIS vs. 20 µM NIMO + 20 µM CIS	-394.2	-2064 to 1275	ns	0.9316
20 µM CIS vs. 20 µM NIMO + 10 µM CIS	-354.7	-2024 to 1315	ns	0.9520
20 µM CIS vs. 20 µM NIMO + 20 µM CIS	-365.5	-2035 to 1304	ns	0.9469
20 µM NIMO + 10 µM CIS vs. 20 µM NIMO + 20 µM CIS	-10.80	-1680 to 1659	ns	>0.9999
pCREB				
control vs. 10 µM CIS	-1629	-27469 to 24211	ns	0.9995
control vs. 20 µM CIS	-9503	-35342 to 16337	ns	0.7462
control vs. 20 µM NIMO + 10 µM CIS	-4755	-30595 to 21085	ns	0.9710
control vs.	-9217	-35057 to 16623	ns	0.7654

20 µM NIMO + 20 µM CIS				
10 µM CIS vs. 20 µM CIS	-7873	-33713 to 17966	ns	0.8484
10 µM CIS vs. 20 µM NIMO + 10 µM CIS	-3126	-28966 to 22714	ns	0.9938
10 µM CIS vs. 20 µM NIMO + 20 µM CIS	-7588	-33428 to 18252	ns	0.8641
20 µM CIS vs. 20 µM NIMO + 10 µM CIS	4747	-21092 to 30587	ns	0.9711
20 µM CIS vs. 20 µM NIMO + 20 µM CIS	285.3	-25555 to 26125	ns	>0.9999
20 µM NIMO + 10 µM CIS vs. 20 µM NIMO + 20 µM CIS	-4462	-30302 to 21378	ns	0.9769

Diff.: Difference; control: solvent control (absolute ethanol, 0.9% NaCl); NIMO: nimodipine; CIS: cisplatin; ns: not significant

a)

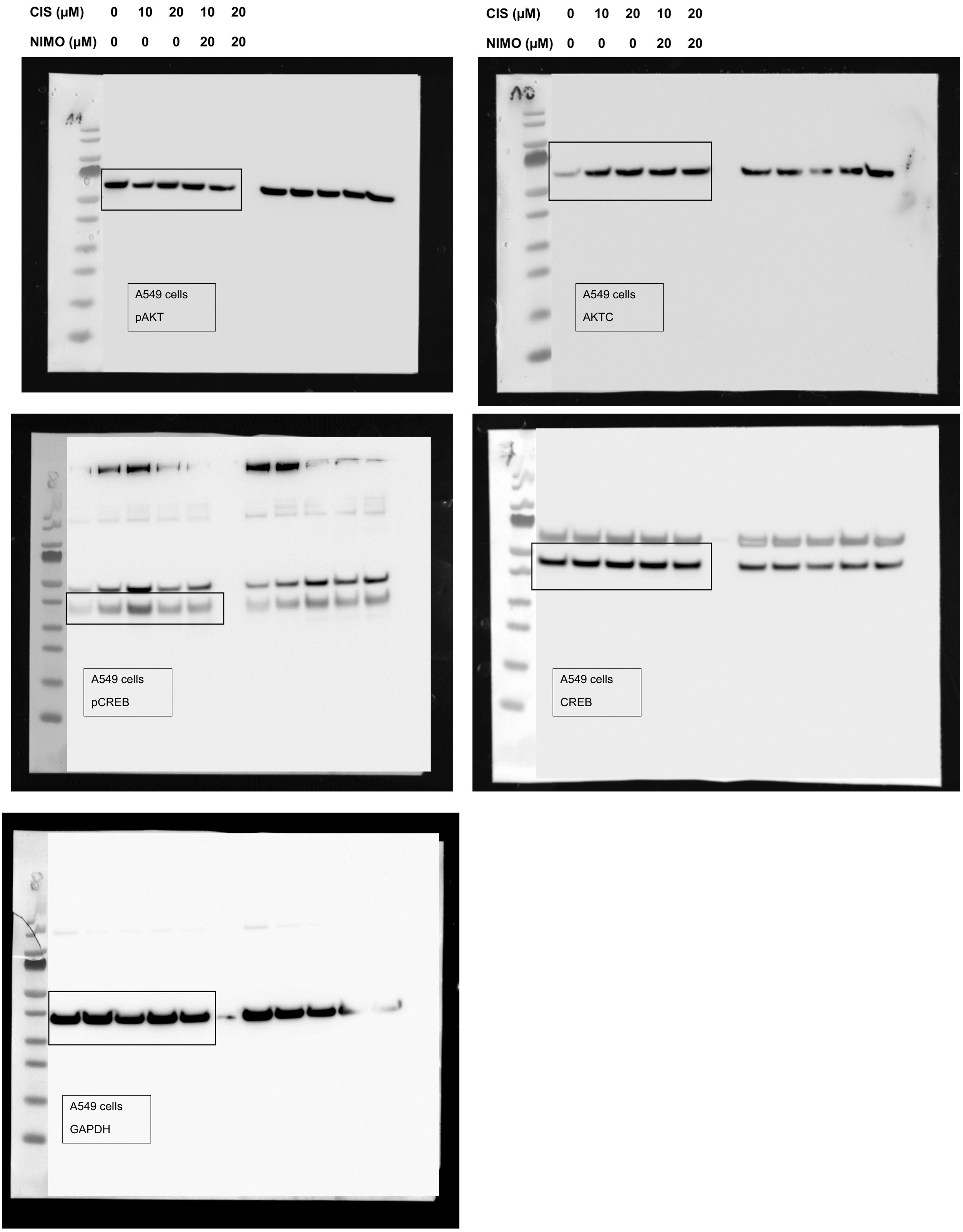

CIS (µM) 0 10 20 10 20
NIMO (µM) 0 0 0 20 20
CIS (µM) 0 10 20 10 20
NIMO (µM) 0 0 0 20 20
A549 cells
pAKT
A549 cells
AKTC
A549 cells
pCREB
A549 cells
CREB
A549 cells
GAPDH

b)

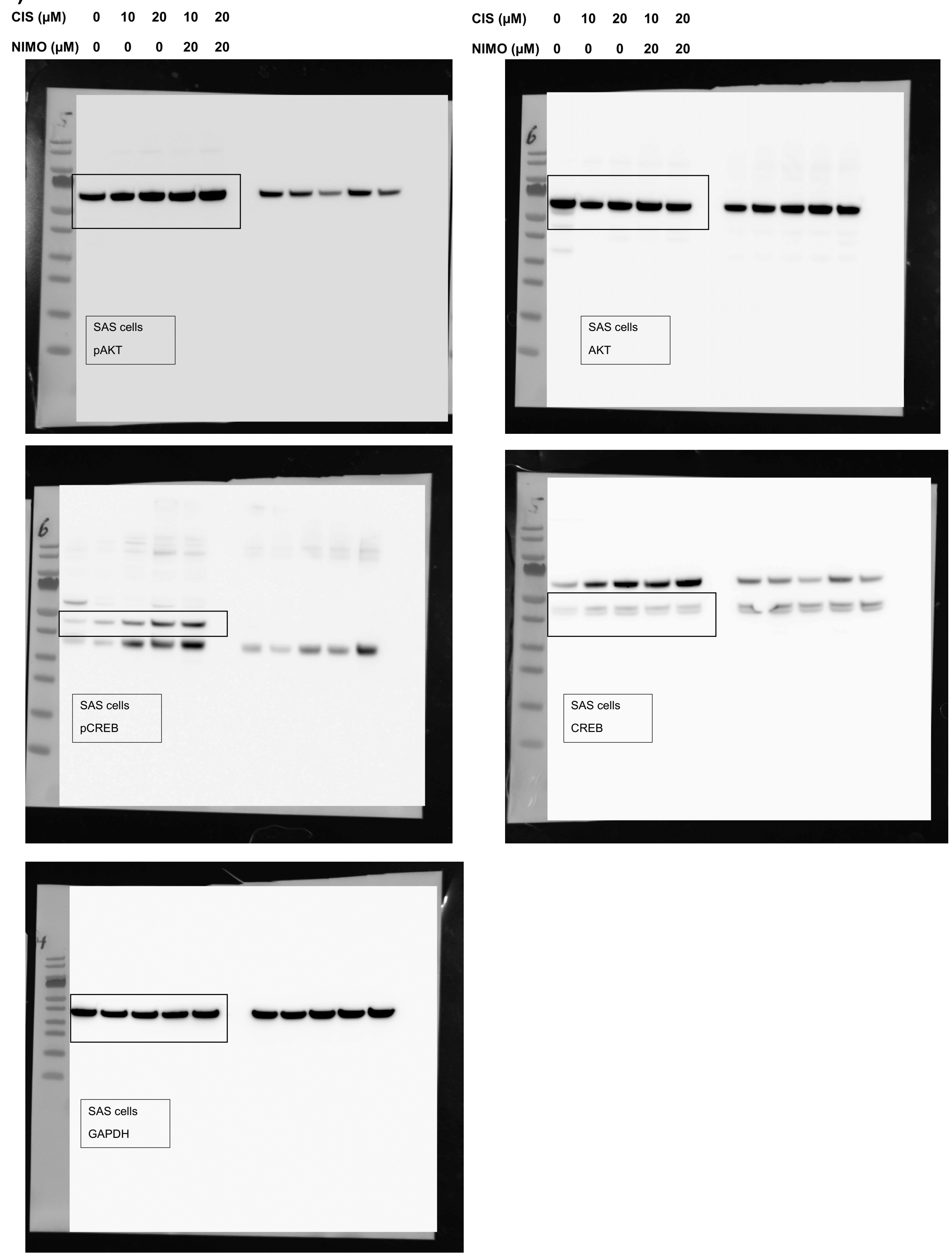
CIS (μM) 0 10 20 10 20
NIMO (μM) 0 0 0 20 20
SAS cells
pAKT
CIS (μM) 0 10 20 10 20
NIMO (μM) 0 0 0 20 20
SAS cells
AKT
SAS cells
pCREB
SAS cells
CREB
SAS cells
GAPDH

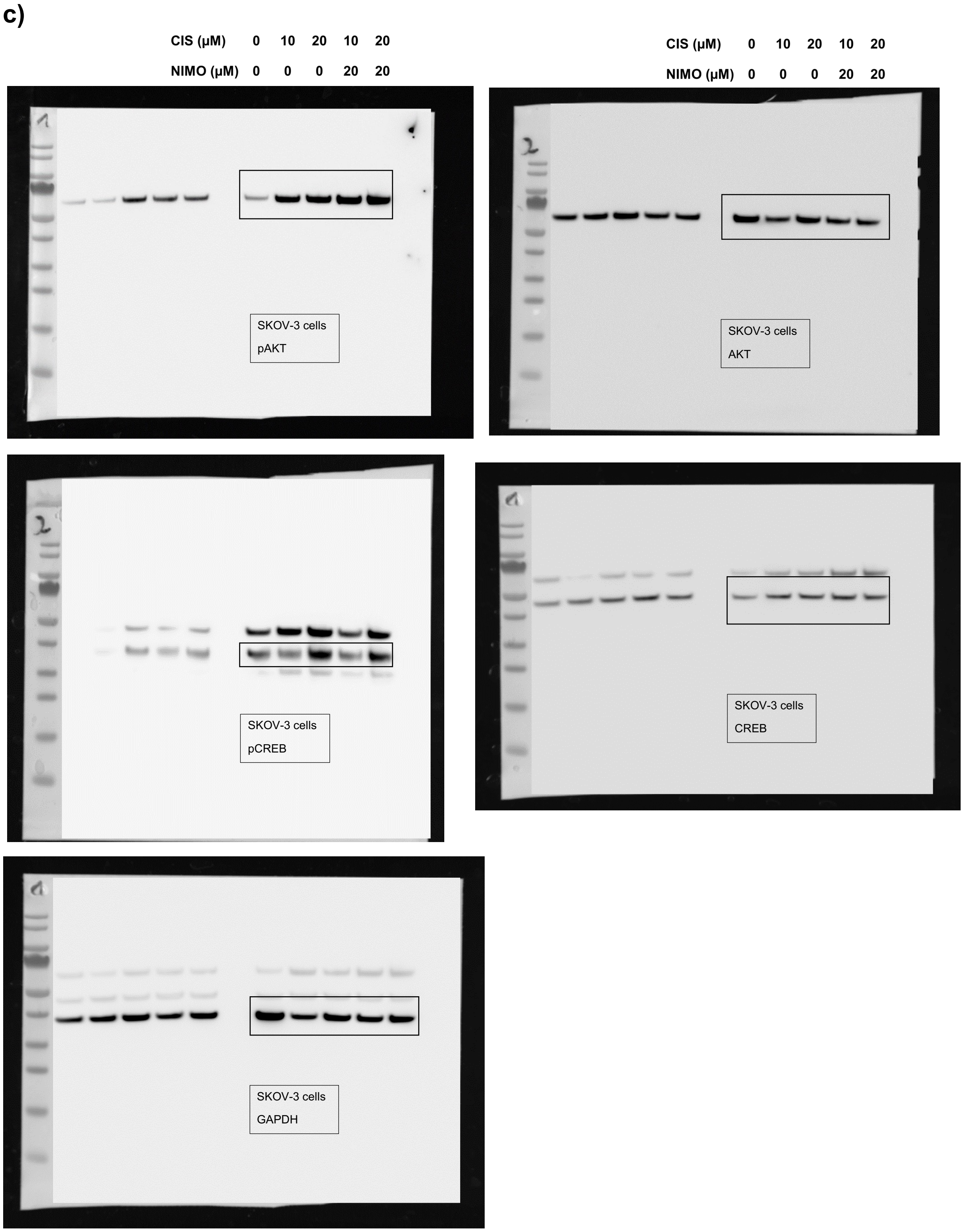

Figure S8: Full-length Western blot membranes shown in Figure 5. The immunoblots were prepared as described in the material and methods section. The antibodies and dilutions used are listed in Table S1. PageRuler (180-10 kDa, #26616, Thermo Fisher Scientific, Waltham, MA, USA) was used as molecular weight marker. Shown are the blots for A549 cells (a), SAS cells (b) and SKOV-3 cells (c).

Table S15. LMO4 level quantification statistical analysis and CI of SW10 cells

**SW10 cells**	*LMO4 protein level [%] Mean Diff.*	*95.00 % CI of diff.*	*summary*	*adjusted p value*
control vs. 10 µM CIS	56.51	32.73 to 80.28	***	0.0001

control vs. 20 µM CIS	82.95	59.17 to 106.7	****	<0.0001
control vs. 20 µM NIMO + 10 µM CIS	46.53	22.76 to 70.31	***	0.0005
control vs. 20 µM NIMO + 20 µM CIS	69.67	45.89 to 93.44	****	<0.0001
10 µM CIS vs. 20 µM CIS	26.44	2.668 to 50.22	*	0.0282
10 µM CIS vs. 20 µM NIMO + 10 µM CIS	-9.974	-33.75 to 13.80	ns	0.6521
10 µM CIS vs. 20 µM NIMO + 20 µM CIS	13.16	-10.61 to 36.94	ns	0.4131
20 µM CIS vs. 20 µM NIMO + 10 µM CIS	-36.42	-60.19 to -12.64	**	0.0036
20 µM CIS vs. 20 µM NIMO + 20 µM CIS	-13.28	-37.06 to 10.49	ns	0.4049
20 µM NIMO + 10 µM CIS vs. 20 µM NIMO + 20 µM CIS	23.13	-0.6402 to 46.91	ns	0.0574

Diff.: Difference; * p ≤ 0.05; ** p ≤ 0.01; *** p ≤ 0.001; **** p ≤ 0.0001; control: solvent control (absolute ethanol, 0.9% NaCl); NIMO: nimodipine; CIS: cisplatin; ns: not significant

Table S16. LMO4 level quantification statistical analysis and CI of RN33B cells

**RN33B cells**	*LMO4 protein level [%] Mean Diff.*	*95.00 % CI of diff.*	*summary*	*adjusted p value*
control vs. 10 µM CIS	68.19	55.18 to 81.21	****	<0.0001
control vs. 20 µM CIS	81.07	68.06 to 94.09	****	<0.0001
control vs. 20 µM NIMO + 10 µM CIS	59.11	46.09 to 72.12	****	<0.0001
control vs. 20 µM NIMO + 20 µM CIS	72.36	59.34 to 85.38	****	<0.0001
10 µM CIS vs. 20 µM CIS	12.88	-0.1391 to 25.90	ns	0.0528
10 µM CIS vs. 20 µM NIMO + 10 µM CIS	-9.087	-22.10 to 3.930	ns	0.2224
10 µM CIS vs. 20 µM NIMO + 20 µM CIS	4.166	-8.852 to 17.18	ns	0.8255
20 µM CIS vs. 20 µM NIMO + 10 µM CIS	-21.97	-34.98 to -8.948	**	0.0018
20 µM CIS vs. 20 µM NIMO + 20 µM CIS	-8.713	-21.73 to 4.305	ns	0.2535
20 µM NIMO + 10 µM CIS vs.	13.25	0.2352 to 26.27	*	0.0456

20 µM NIMO +<br>20 µM CIS				

Diff.: Difference; * p ≤ 0.05; ** p ≤ 0.01; **** p ≤ 0.0001; control: solvent control (absolute ethanol, 0.9% NaCl); NIMO: nimodipine; CIS: cisplatin; ns: not significant

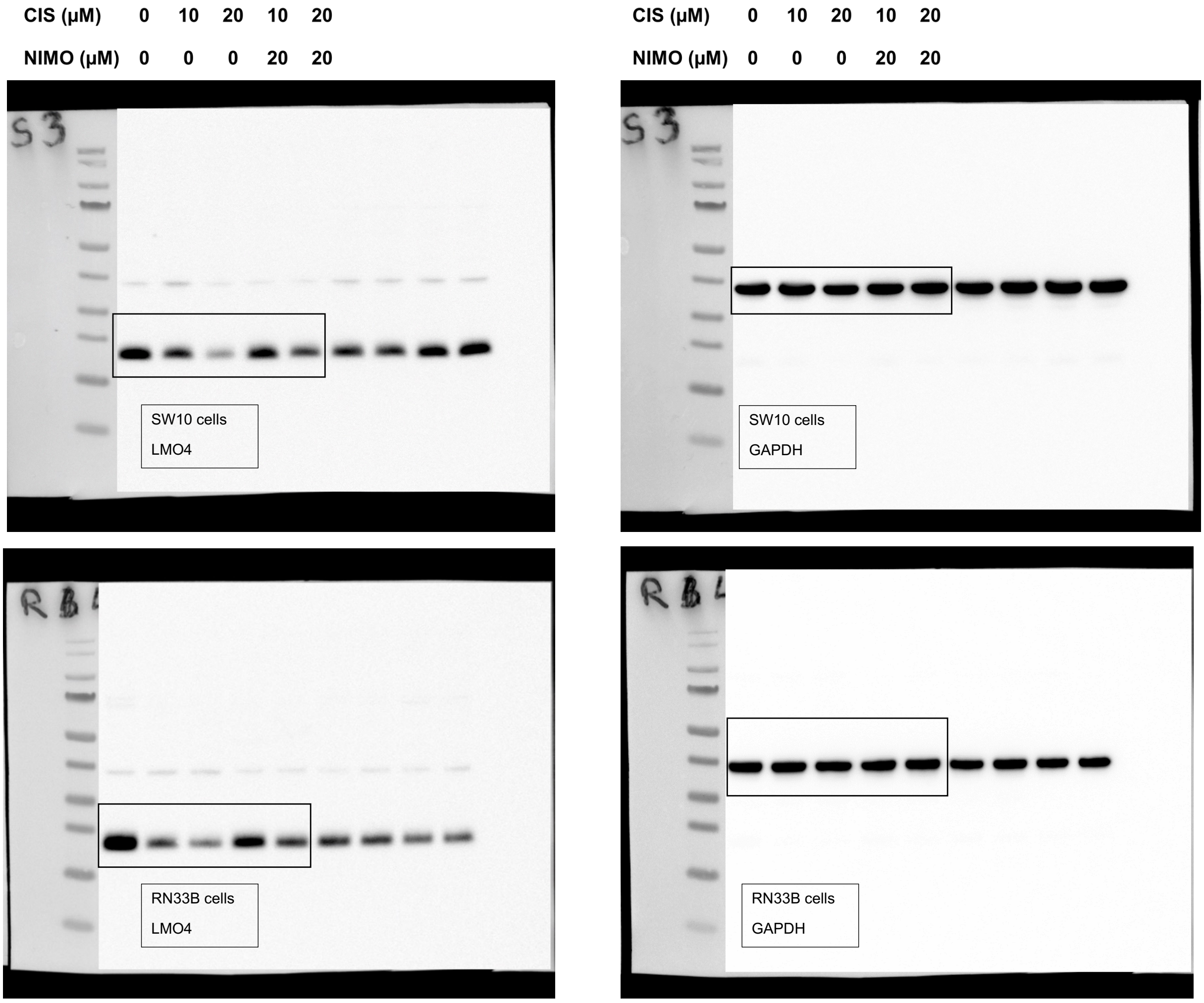

Figure S9: Full-length Western blot membranes shown in Figure 6. The immunoblots were prepared as described in the material and methods section. The antibodies and dilutions used are listed in Table S1. PageRuler (180-10 kDa, #26616, Thermo Fisher Scientific, Waltham, MA, USA) was used as molecular weight marker.

Table S17. LMO4 level quantification statistics and CI of A549 cells

**A549 cells**	*LMO4 protein level [%] Mean Diff.*	*95.00 % CI of diff.*	*summary*	*adjusted p value*
control vs.<br>10 µM CIS	12.19	-70.49 to 94.87	ns	0.9870
control vs.<br>20 µM CIS	63.71	-18.97 to 146.4	ns	0.1579
control vs.<br>20 µM NIMO +<br>10 µM CIS	22.27	-60.41 to 105.0	ns	0.8955
control vs.<br>20 µM NIMO +<br>20 µM CIS	58.58	-24.10 to 141.3	ns	0.2119
10 µM CIS vs.<br>20 µM CIS	51.52	-31.16 to 134.2	ns	0.3106
10 µM CIS vs.<br>20 µM NIMO +<br>10 µM CIS	10.08	-72.60 to 92.76	ns	0.9936

10 µM CIS vs. 20 µM NIMO + 20 µM CIS	46.39	-36.29 to 129.1	ns	0.4012
20 µM CIS vs. 20 µM NIMO + 10 µM CIS	-41.44	-124.1 to 41.24	ns	0.5019
20 µM CIS vs. 20 µM NIMO + 20 µM CIS	-5.134	-87.81 to 77.55	ns	0.9995
20 µM NIMO + 10 µM CIS vs. 20 µM NIMO + 20 µM CIS	36.31	-46.37 to 119.0	ns	0.6156

Diff.: Difference; control: solvent control (absolute ethanol, 0.9% NaCl); NIMO: nimodipine; CIS: cisplatin; ns: not significant

Table S18. LMO4 level quantification and CI of SAS cells

**SAS cells**	*LMO4 protein level [%] Mean Diff.*	*95.00% CI of diff.*	*summary*	*adjusted p value*
control vs. 10 µM CIS	93.72	86.83 to 100.6	****	<0.0001
control vs. 20 µM CIS	95.43	88.54 to 102.3	****	<0.0001
control vs. 20 µM NIMO + 10 µM CIS	94.01	87.13 to 100.9	****	<0.0001
control vs. 20 µM NIMO + 20 µM CIS	96.00	89.11 to 102.9	****	<0.0001
10 µM CIS vs. 20 µM CIS	1.710	-5.176 to 8.596	ns	0.9194
10 µM CIS vs. 20 µM NIMO + 10 µM CIS	0.2957	-6.590 to 7.182	ns	0.9999
10 µM CIS vs. 20 µM NIMO + 20 µM CIS	2.278	-4.608 to 9.164	ns	0.8085
20 µM CIS vs. 20 µM NIMO + 10 µM CIS	-1.414	-8.300 to 5.472	ns	0.9573
20 µM CIS vs. 20 µM NIMO + 20 µM CIS	0.5681	-6.318 to 7.454	ns	0.9986
20 µM NIMO + 10 µM CIS vs. 20 µM NIMO + 20 µM CIS	1.982	-4.904 to 8.869	ns	0.8719

Diff.: Difference; **** p ≤ 0.0001; control: solvent control (absolute ethanol, 0.9% NaCl); NIMO: nimodipine; CIS: cisplatin; ns: not significant

Table S19. LMO4 level quantification statistics and CI of SKOV-3 cells

**SKOV-3 cells**	*LMO4 protein level [%] Mean Diff.*	*95.00 % CI of diff.*	*summary*	*adjusted p value*
control vs. 10 µM CIS	77.74	53.56 to 101.9	****	<0.0001
control vs. 20 µM CIS	95.70	71.52 to 119.9	****	<0.0001
control vs.	82.51	58.33 to 106.7	****	<0.0001

20 µM NIMO + 10 µM CIS				
control vs. 20 µM NIMO + 20 µM CIS	96.64	69.61 to 123.7	****	<0.0001
10 µM CIS vs. 20 µM CIS	17.96	-6.220 to 42.14	ns	0.1745
10 µM CIS vs. 20 µM NIMO + 10 µM CIS	4.770	-19.41 to 28.95	ns	0.9596
10 µM CIS vs. 20 µM NIMO + 20 µM CIS	18.90	-8.135 to 45.93	ns	0.2137
20 µM CIS vs. 20 µM NIMO + 10 µM CIS	-13.19	-37.37 to 10.99	ns	0.4120
20 µM CIS vs. 20 µM NIMO + 20 µM CIS	0.9392	-26.09 to 27.97	ns	>0.9999
20 µM NIMO + 10 µM CIS vs. 20 µM NIMO + 20 µM CIS	14.13	-12.91 to 41.16	ns	0.4496

Diff.: Difference; **** $p \leq 0.0001$; control: solvent control (absolute ethanol, 0.9% NaCl); NIMO: nimodipine; CIS: cisplatin; ns: not significant

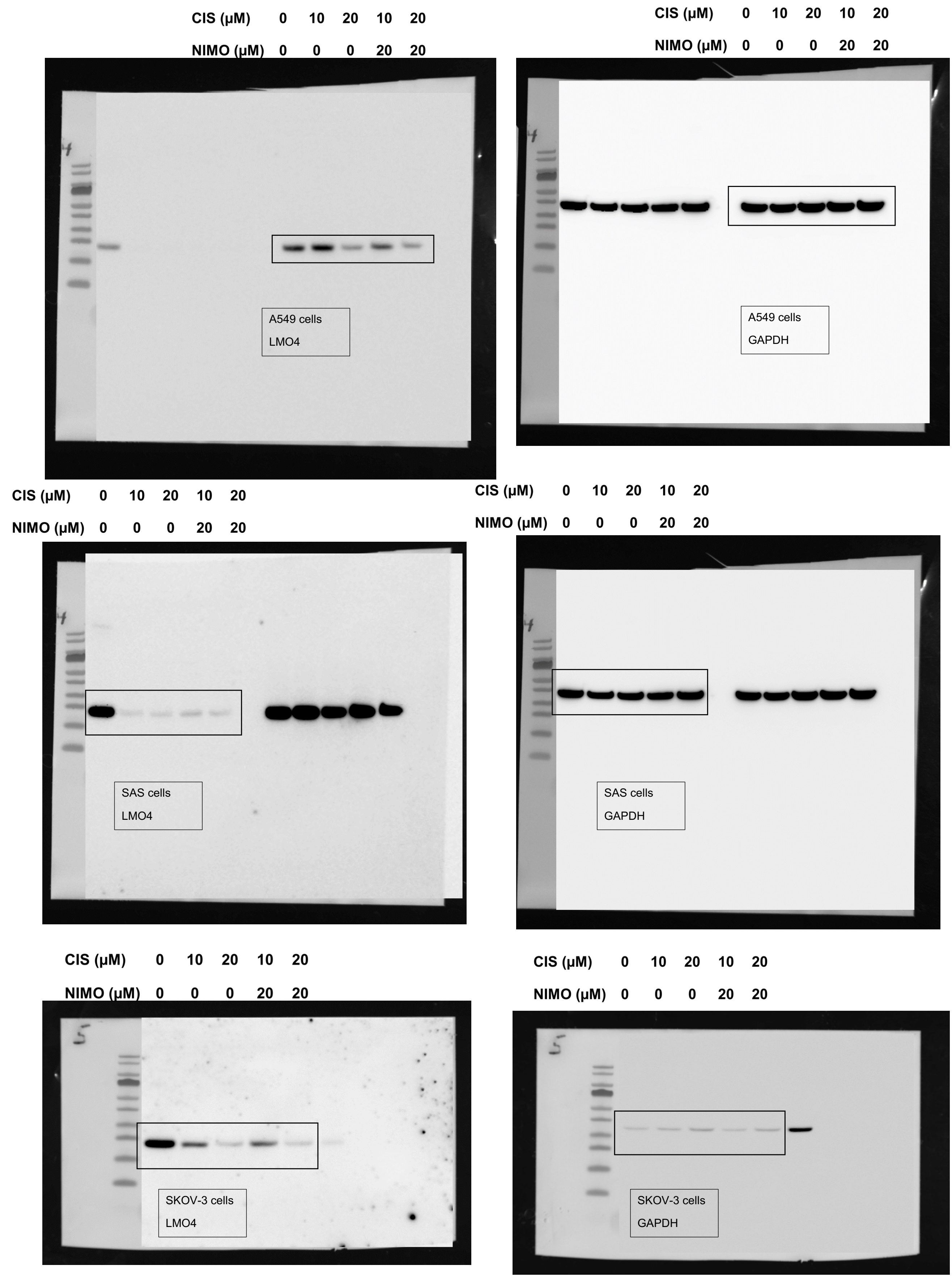

Figure S10: Full-length Western blot membranes shown in Figure 7. The immunoblots were prepared as described in the material and methods section. The antibodies and dilutions used are listed in Table S1. PageRuler (180-10 kDa, #26616, Thermo Fisher Scientific, Waltham, MA, USA) was used as molecular weight marker.
